# High-resolution Bayesian chronology of the earliest evidence of domesticated animals in the Dutch wetlands (Hardinxveld-Giessendam archaeological sites)

**DOI:** 10.1371/journal.pone.0280619

**Published:** 2023-01-24

**Authors:** Merita Dreshaj, Michael Dee, Nathalie Brusgaard, Daan Raemaekers, Hans Peeters

**Affiliations:** 1 Groningen Institute of Archaeology, University of Groningen, Groningen, Netherlands; 2 Centre for Isotope Research, Energy Academy, Groningen, Netherlands; New York State Museum, UNITED STATES

## Abstract

The archaeological sites of Hardinxveld-Giessendam de Bruin and Polderweg, situated in the Rhine-Meuse delta, are the best-preserved Mesolithic sites in the Netherlands. Due to the early appearance of domesticated animals in their faunal assemblage, they are also integral to the research of the emergence of animal husbandry in the region. This study focuses on the precise chronology of the sites, using radiocarbon dating and Bayesian modelling of both newly acquired and legacy radiocarbon dates. To mitigate the risk of erroneous dates, we dated the bone collagen of 26 herbivorous and one aquatic mammals from clear archaeological contexts and discovered that the most recent occupational phases at both sites are several centuries younger than previously thought. This is consistent with material evidence of lifestyle changes in the final phase at Hardinxveld-Giessendam de Bruin, which is now, according to our chronology, contemporaneous with the similar patterns produced in the region.

## Introduction

In the Low Countries, the fifth millennium BCE sees several remarkable changes with the emergence of pottery production, animal husbandry, and cereal cultivation in the Swifterbant culture [1–5]. In view of the socio-cultural and economic implications of these changes, much discussion centers around their timing and the duration of transitions, and several models have been proposed. Because these novelties occurred over the entire fifth millennium BCE, the prevailing interpretation is that of a long and gradual transition to agriculture [6], initiated by the interaction with LBK farmers from 5300 BCE onwards. In contrast, a ‘short early’ model [2] propagates the idea of farmer settlements in coastal areas, while inland wetland sites primarily represent hunter-gatherer-fisher stations. Any evidence of coastal farming sites would have been completely lost, owing to erosion. Thus, this transition could have occurred considerably sooner than previously thought, a hypothesis which has been criticized for its oversimplification of settlement systems [7]. The third hypothesis, the ‘short late’ model, proposes an introduction of animal husbandry and cereal cultivation in north-western Europe around approximately 4000 BCE, coinciding with similar patterns in Scandinavia and the British Isles [8, 9].

Notwithstanding the explicit evidence of crop cultivation in the Dutch wetlands [10, 11], reconstructing the timeline of animal husbandry remains ambiguous due to the difficulties in determining the domestic status of locally available species (*Sus* and *Bos*) without multi-proxy research [4, 12, 13]. Moreover, and central to the research presented here, there is a lack of direct dates on animals for which the domesticated status has been established. There is also much uncertainty about the chronological phasing of key sites. Additionally, the narrative of the fifth millennium is hampered by legacy datasets replete with untrustworthy radiocarbon results, and a calibration plateau nested in the final quarter of the millennium that causes absolute dates to extend across multiple centuries [14]. The question is how to overcome these shortcomings, which have led to rather blurred narratives about the establishment of farming societies in the Low Countries.

**Fig 1:**
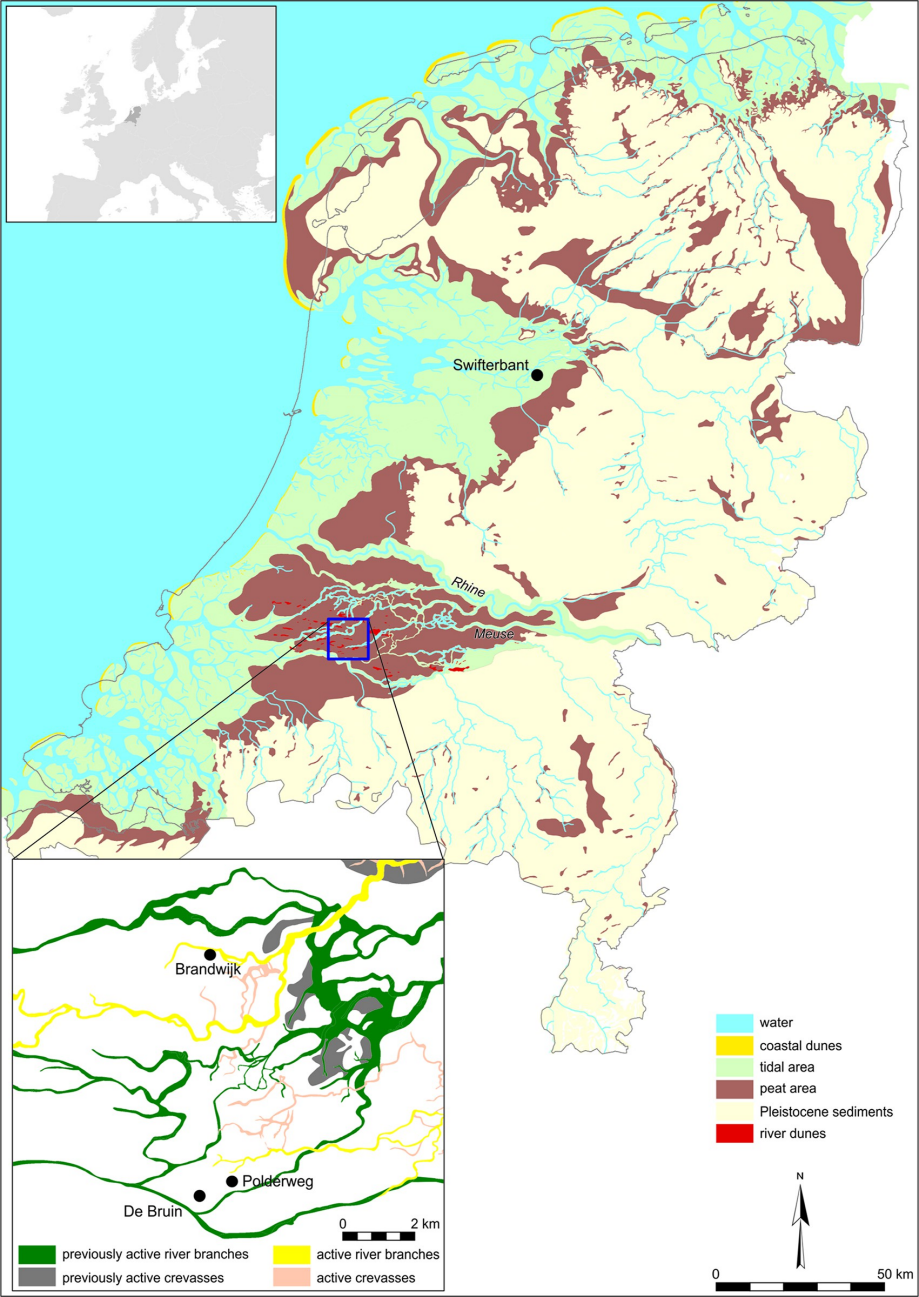
Palaeographical map of the Netherlands (3850 BCE) with location of the Rhine-Meuse estuary in relation to the Swifterbant sites in Flevoland [23, 24] referred in the text. Insert: local palaeogeographical map with locations of Hardinxveld-Giessendam (Polderweg and De Bruin) and Brandwijk Kerkhof sites. [After Vos et al. [25]].

With the possibility of obtaining high-precision radiocarbon dates, the benefits of improved sample preparation, and the availability of powerful Bayesian modelling tools, radiocarbon datasets can now be analyzed more effectively, and can make a significant contribution to the debate. Such chronological modeling is an important aspect of the project ‘The Emergence of Domestic Animals in the Netherlands’ (EDAN), based at the University of Groningen [15]. On this project, we focus on a series of wetland sites dating to the fifth millennium BCE. Among these, the sites of Hardinxveld-Giessendam Polderweg (hereafter ‘Polderweg’) and Hardinxveld-Giessendam de Bruin (hereafter ‘De Bruin’) are key to the understanding of the Late Mesolithic and Early Neolithic periods in the Netherlands [Fig 1]. These sites feature the oldest Swifterbant pottery, the earliest remains of domesticated animals (sheep/goat, small-sized *Sus* and *Bos*), and possible evidence of dairy production [16], while the dwellers retained a lifestyle where hunting and gathering were the primary subsistence strategies (the broad spectrum economy [13, 16–19]). The presence of domesticated *Sus* and/or *Bos* was explored through morphometric and stable isotope analysis [15] and is currently undergoing aDNA study [Erven et al. in prep.]. Although the stable isotope analysis did not elucidate any evidence of dietary management or harvesting practices indicative of animal husbandry, it is clear that these sites provide information about communities that were familiar with livestock rearing (sheep/goat, small-sized pigs and cattle) and, if anything, their practices likely preceded animal husbandry [15]. Consequently, Hardinxveld sites provide complementary information on the chronological framework within which the emergence of animal husbandry in the Dutch wetlands occurred.

While the chronology of these sites was considered resolved [20–22], a recent re-evaluation of legacy radiocarbon data from De Bruin led to the conclusion that its chronostratigraphy lacks reliable data for a convincing chronological interpretation [14]. In this paper, we report the outcomes of a high-precision chronological study, aimed at resolving this issue. We obtained radiocarbon dates on 27 bone collagen samples from targeted contexts across all habitation phases of both sites. To avoid reservoir effects, we limited the sample choice to herbivorous mammals, namely *Bos* (aurochs/cattle), *Cervus elaphus* (red deer), *Sus* (wild boar with herbivorous diet, according to the recent stable isotope study [15]), and *Castor fiber* (Eurasian beaver). For an insight into the reservoir effect, we dated a single specimen of *Lutra lutra* (otter), as well. Furthermore, to obtain the most precise and reliable date ranges possible, we used Bayesian statistics to combine our new dates with carefully screened legacy dates in chronological models. The result is a new chronological framework for both sites, which lays the groundwork for addressing uncertainty in the chronology of the Mesolithic-Neolithic transition in the Dutch wetlands.

## Site context and description

De Bruin and Polderweg are located close to each other (1 km) on the top of Pleistocene river dunes in the swampy Rhine-Meuse estuary [Fig 1]. Both sites were Late Mesolithic camps occupied in largely contemporaneous phases for a millennium, featuring remains attributed to the Swifterbant culture [17, 26]. Because of the resemblance in material culture and geoarchaeological context, alongside their proximity, these sites are considered ‘twin sites’, likely used by cooperating groups or the same community, possibly with alternating site functions [27].

Zooarchaeological and archaeobotanical evidence suggests that they were winter camps at first, turning into year-round camps from 5000 BCE onwards, although they were never continuously occupied [18, 28–30]. During the occupation of these dunes, the surrounding environment became increasingly wet due to Holocene sea-level rise which affected the lower Rhine-Meuse floodplain vegetation, as evidenced by the extension of alder carr [29] and indications of a water-table rise in the stratigraphy of both sites [20, 21]. Polderweg was overgrown by marshland first, followed by De Bruin some centuries later.

It must be noted that hydrological dynamics within lowland floodplains can vary a lot, even at smaller (sub)regional scale, notably due to beaver activity which can have a major impact on local water levels, an extension of marshland, and on sedimentary regimes [31–33]. As is the case within the Polderweg and De Bruin area, lower order river gullies (side gullies; crevasse gullies; tributaries) will more easily show variability in local dynamics, compared to main river systems directly connected to the sea and tidal regimes. In fact, the site investigators consider the local groundwater level curve to be incorrect when applied to Hardinxveld sites. The Polderweg data suggest that groundwater levels must have been around 80 cm higher there than at De Bruin, at the same time, something which the same investigators considered an impossibility [20, 21].

Typically, the wider stratigraphical contexts of Polderweg and De Bruin are characterised by complex vertical and lateral sequences of sediments (peat; detritus; clay; sand) that represent various geomorphological entities and sedimentary environments [20, 21, 34, 35]. Just how various (individual) stratigraphical units are chronologically related to phases of human occupation on the river dunes, appears difficult to establish, other than in broad terms.

According to the archaeological evidence, both sites reflect a subsistence pattern typical for hunter-gatherer-fisher communities, mainly of beavers, otters and suids, seemingly processed on site [18, 28]. Remains of *Bos* (auroch/cattle) are present in strikingly small numbers and, at Polderweg, only as artefacts. In the last phase of De Bruin, several remains of domestic sheep/goat (*Ovis aries*/*Capra hircus)* and *Sus* and *Bos* akin in size to domestic pigs and cattle appear in the faunal assemblage [18, 28]. A recent zooarchaeological and stable isotope study on these sites ruled out dietary and population management of *Sus* and *Bos* species that would reflect animal husbandry. Indeed, it was proposed that the absence of neonates and young piglets in this phase may suggest that these small-sized remains were not husbanded but were the product of exchange with farmers, a pattern documented in similar contexts elsewhere, and which also precedes animal husbandry [15, 36, 37]. In this context, wild species remain dominant subsistence strategy, with a special emphasis on hunting beavers, which increases towards the last occupation phases [18, 28]. Thus, other than the size of the bones, there has not yet been conclusive proof of domestic pigs or cattle or compelling evidence of animal husbandry at the site [13, 15, 18].

### Stratigraphy and chronology of Polderweg

Excavations at Polderweg revealed large pits, hearths, postholes (likely from dwellings), and several human and dog burials intersected by felled trees that were assumed to be connected to anthropogenic activity. The excavated area largely represents the sloping periphery of the settlement, where activities like fishing, butchering, tool manufacturing and (waste) disposal were carried out. The habitational area with dwellings will have shifted to higher parts of the sand dune with rising (ground)water tables [20]. Large pits and postholes in the oldest phases (0 and 1) may represent dwelling structures with sunken floors [38]. The site is interpreted as a basecamp, implying a relatively long and intensive use of space, [20] which will have led to disturbance of the subsoil and mixing and blurring of remains from various occupational phases, i.e. palimpsest formation [39].

**Fig 2:**
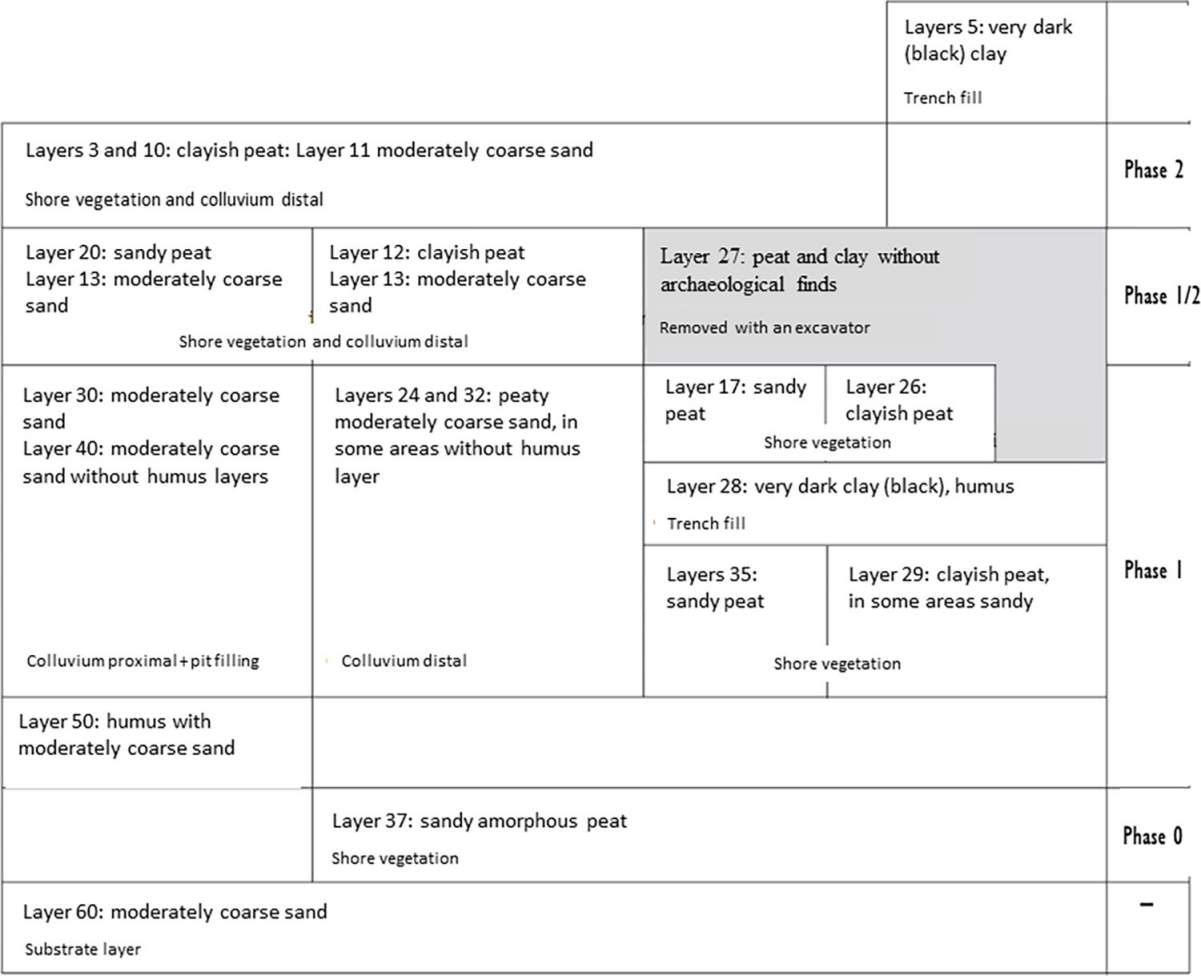
Overview of the stratigraphy of Polderweg. The archaeological layers are denoted by numbers. The right horizontal pane marks the occupational phases encompassing respective archaeological layers. Figure from Mol & Louwe Kooijmans, [20], p.56, Fig 3.1.; translated.

The stratigraphy of the site was divided into three main occupational phases, with an additional phase 0 corresponding to layer 37 [Fig 2], often interpreted as the lowermost part of phase 1 [20]. In layer 37, the remains of a sunken-floor hut next to a modest fireplace were uncovered, as well as four postholes and the burial of an adult woman. The succeeding phase 1 corresponds to more intense human activity, as evidenced by denser distributions of artefacts and indications of heavier built structures, and more hearths. This phase ties in with increased colluviation paralleled by local groundwater rise. Colluviation decreases significantly in the following phases 1/2 and 2 when the dry area became reduced to the top of the dune and evidence for human activity diminishes within the excavated area because excavation trenches covered only the peripheral zone. Unsurprisingly, these phases are characterized by much less archaeological material. For example, phase 2 is attested by only a single concentration of bone and charcoal deposited in, or on top of a sandy layer. These remains were found amidst numerous tree trunks, one of which was dendrochronologically dated to 4900 BCE, and served as an anchor for the chronology of the last phase, despite its unclear connection to human occupation. Because this layer is sandy, all evidence of potentially built structures has been lost [20]. As a result, the reconstruction of the occupancy pattern for phases 1/2 and 2 must be approached with caution.

The chronology, as interpreted in the excavation report, was established through a series of radiocarbon dates collected across the occupation phases during the excavation. The material dated ranged from short-lived plant material to bone collagen on bones of omnivorous species (including human burials), charred food residues on pottery, wood, and charcoal ([20], Table 1 in S1 File). As a result, more than half of the radiocarbon dataset potentially consisted of incorrect dates due to the freshwater reservoir and old wood effects.

### Stratigraphy and chronology of De Bruin

Unlike Polderweg, where most of the archaeological remains are produced from phase 1, De Bruin allows for the sequence of phases to be studied in more detail. On this site, various types of features were uncovered, namely artefact concentrations, waste pits, postholes, two human burials, one animal burial, and a potential canoe landing stage [40]. Similar to Polderweg, the function of the site remains unclear and may have changed over time.

**Fig 3:**
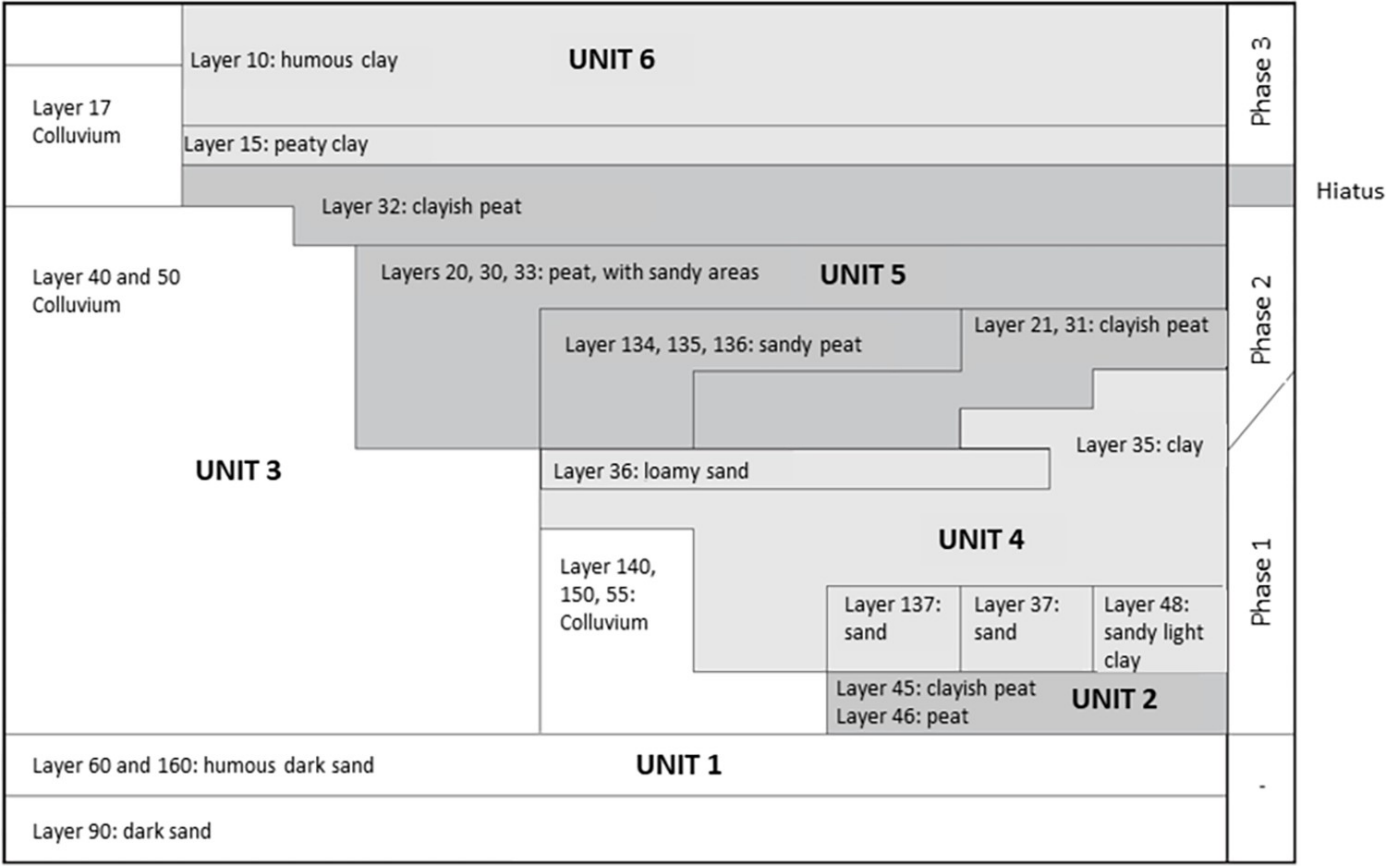
Overview of the stratigraphy of De Bruin. The archaeological layers are placed within six lithostratigraphic units. The horizontal pane on the right marks the three occupational phases encompassing the respective archaeological layers. Figure from Mol & Louwe Kooijmans, 2001 [21], p.62, Fig 3.3.; translated.

The stratigraphy is divided into six lithostratigraphic units comprising 28 archaeological layers. The archaeological layers were grouped into three occupational phases divided by hiatuses [Fig 3]. While most layers were assigned to a single phase, some stretched across two phases or had an uncertain attribution, such as colluvium layers 40 and 50. Similarly, layer 32 extends across the end of phase 2, the hiatus, and the start of phase 3. In addition, layer 35 was divided between phases 1 and 2, with a noticeable hiatus between them [21].

A recent assessment and Bayesian analysis of De Bruin’s radiocarbon dataset revealed that more than half of the dates are most likely erroneous, owing to either the freshwater reservoir effect or the inbuilt age [14]. This outcome is largely due to the diversity of sampled material, as is the case with Polderweg. The remaining dates were obtained on short-lived material, but in several circumstances were susceptible to context misassociation (Table 1 in S1 File) [13, 14, 21]. The model results indicated an urgent need for more reliable dates, particularly for the latter two occupational phases. Stratigraphic difficulties were predicted, given that the excavated area of the site had been affected by erosion, colluviation and multiple reuses of habitational surfaces. However, the earliest phase of the dwelling camp, located on the lower slopes of the dune, was more rapidly concealed by sediment, allowing for greater stratigraphic preservation [21]. This is why the chronological interpretations of this site are so diverse and in need of further study [14].

## Materials and methods

The permit to sample animal remains (archaeological bones) used in this study was obtained from the ‘Provinciaal Archeologisch Depot Zuid-Holland’ (Provincial Archaeological Depot of South Holland, Alphen aan de Rijn, the Netherlands). Permit Bruikleennr. 2020–1.

Our method comprised three interrelated steps: 1. evaluating legacy data using the principles of chronometric hygiene; 2. radiocarbon dating bone collagen from predominantly herbivorous mammals throughout all occupational periods; and 3. analyzing both the new and legacy dates in Bayesian models in the program OxCal [41].

### Chronometric hygiene

’Chronometric hygiene’ refers to the systematic revision of radiocarbon datasets to distinguish dates based on the reliability of the dated material, archaeological context and supporting analytical data [42, 43]. Following this approach, we divided the dates into three major categories, based on material type. The first grade [optimal sample material] accounts for less than half of the dataset, with all of the remaining dates potentially subject to either a freshwater reservoir effect or inbuilt age (See Table 1). In a recent revision of the chronology of De Bruin that employed Bayesian modeling, samples susceptible to the "old wood effect" such as charcoal and wood, were compared with short-lived plant samples from the same context, and similar results were produced [14]. As a result, we decided to denote them as second grade, distinguishing charcoal and wood from samples likely affected by the freshwater reservoir effect (third grade). The latter, however, appeared to result in offsets that were both unpredictable and not easily explicable. A similar pattern is evident in other radiocarbon datasets in the region [14].

**Table 1:** Classification of legacy dates based on their degree of reliability: Polderweg & de Bruin.

Grade	(Possible) Offset	The material present in the dataset	Polderweg	De Bruin
**First grade**	none	charred & uncharred seeds	5	11
**Second grade**	old wood effect	charcoal, wood	3	4
**Third grade**	freshwater reservoir effect	bones of omnivores, aquatic animals, animals feeding on aquatic sources, charred residue on pottery	6	7

Following the screening of legacy dates based on material type, we scrutinized the contextual information of the selected dates. The purpose was to understand the relationship between the dated material and the age of deposition of the respective layer. As discussed above, both sites are situated on floodplains and stagnant water bodies, with cases of colluviation or downward movement of material across the slopes of the dunes which often displaces material between phases [20, 21, 34, 35]. This is why it is important to strive for low-movement-environments, such as clay and peat, where stratigraphy is more intact, as was outlined in the report [20–22]. Furthermore, erosion and palimpsest-like stratigraphy [39] may lead to blurred boundaries between anthropogenic layers, imposing the risk of erroneous allocation of dated samples.

Short-lived plant material, or more precisely fast-maturing, reproductive parts of plants (seeds, fruits, nuts) [44], while categorized as an optimal sample for radiocarbon dating, can still return results incongruent with their contexts. In order to tackle this, taxonomic identification is key. It is also important to know whether the sample came from a bulk or a single isolated material. If sampled in bulk, there is risk of intrusive material. Lastly, ascertaining anthropogenic factors in the deposition of plant remains is crucial when reconstructing a timeline of human occupation. Uncharred material, for example, can be related to non-anthropogenic processes which can distort the estimated time ranges of occupation [45].

In the legacy data from Hardinxveld, a large portion of the first-grade samples are macroremains that are either uncharred, or came from bulk samples or have no taxonomic identification (see Table 1 in S1 File). Most of these samples were entrapped within an anthropogenic context but for some dates, collected from the beginning or end of occupation phases, there is no certainty of their anthropogenic origin. Indeed, in excavation reports [20, 21], these alleged *terminus ante quem* and *terminus post quem* dates are interpreted as part of the occupation phases, yet in the first Bayesian modeling attempt, the same dates are presented as unrelated to archaeological layers [22]. Because of the complexity of such circumstances, we address the taphonomic and contextual factors of each contextually incongruent radiocarbon date in the following text and maintain the chronometric classification based on materiality.

### New radiocarbon dating

Following the zooarchaeological and stable isotope study of the faunal assemblage from Hardinxveld Giessendam sites [15], in and taking into account the geological processes at these sites, we sampled the bones of 29 herbivorous and one aquatic mammal from archaeological layers at the beginning, middle, and end of occupational phases, relying on the original stratigraphic interpretation [20, 21], (see Table 2 for more details). This allowed us to be consistent with the unbiased contextualization of newly sampled material and, thanks to the stable isotope study, certain of the herbivorous diet of most of the selected specimens [15]. The aquatic specimen (*Lutra lutra*) allowed us to have an insight into the reservoir effect in the faunal assemblage.

**Table 2:** Radiocarbon dates from bone collagen of herbivorous mammals (exception: GrM-22782) from Polderweg and De Bruin. Sample pretreatment and analysis were carried out at the Centre for Isotope Research, Groningen. Sign ‘*’marks extremely low collagen yield: 0.55% (GrM-23703) and 0.49% (GrM-23514).

Site	sample no.	EDAN no.	sample no.	material	archaeological layer	Archaeological phase	^14^C age (yr BP)	^14^C age uncertainty (±1σ)	C:N ratio	δ^13^C (‰, VPDB)	δ^15^N (‰, Air)	species	element
Polderweg	GrM-22774	EDAN0040	98POLV020526	collagen	37	0	6473	29	3.26	-22.79	5.67	*Sus*	*maxilla*
	GrM-25035	EDAN0238	98POLV020527	collagen	37	0	6492	29	3.30	-22.90	5.46	large mammal [exact species unknown–herbivore due to stable isotope values]	*cranium*
	GrM-25033	EDAN0047	98POLV023327	collagen	28	1	6502	30	3.20	-22.24	5.20	*Sus*	*cranium*
	GrM-22770	EDAN0044	98POLV024294	collagen	29	1	6483	22	3.17	-22.13	4.81	*Sus*	*mandibula*
	GrM-22772	EDAN0045	97POLV008237	collagen	28	1	6298	29	3.22	-23.06	6.12	*Sus*	*mandibula*
	GrM-22774	EDAN0053	97POLV015327	collagen	35	1	6420	29	3.26	-20.67	5.84	*Sus*	*mandibula*
	GrM-22782	EDAN0127	98POLV020165	collagen	32	1	7200	40	3.20	-24.48	11.05	*Lutra lutra*	*humerus*
	GrM-23704	EDAN0115	98POLV020267	collagen	28	1	6550	30	3.21	-22.53	3.70	*Cervus elaphus*	*femur*
	GrM-25036	EDAN0219	98POLV025084	collagen	12	1/2	6017	29	3.20	-22.46	4.20	*Cervus elaphus*	antler
	GrM-26626	EDAN0245	97POLV013649	collagen	20	1/2	6167	29	3.20	-21.79	3.63	*Sus*	*femur*
	GrM-25037	EDAN0241	98POLV020145	collagen	20	1/2	6223	29	3.20	-21.00	6.05	*Sus*	*ulna*
	GrM-25044	EDAN0148	97POLV004033	collagen	10	2	5905	29	3.20	-21.66	6.60	*Sus*	*tibia*
	GrM-25045	EDAN0126	97POLV000481	collagen	5	2	5841	29	3.20	-22.20	5.19	*Castor fiber*	*femur*
	GrM-23741	EDAN0147	97POLV002865	collagen	10	2	5889	27	3.30	-21.98	5.67	*Sus*	*femur*
	GrM-23514*	EDAN0134	97POLV010457	collagen	29	1	5383	26	3.10	-22.30	3.67	*Sus*	*mandibula*
De Bruin	GrM-23703*	EDAN0110	98BRUV020153	collagen	1017	?	6345	28	3.41	-21.63	5.48	*Sus*	*mandibula*
	GrM-22851	EDAN0013	98BRUV011267	collagen	48	1	6319	29	3.23	-21.64	5.59	*Sus*	*mandibula*
	GrM-22778	EDAN0033	98BRUV013488	collagen	46	1	6425	50	3.53	-22.73	5.59	*Sus*	*mandibula*
	GrM-25040	EDAN0010	98BRUV010491	collagen	134	2	6126	29	3.10	-20.58	3.57	*Sus*	*mandibula*
	GrM-25043	EDAN0008	98BRUV019285	collagen	134	2	6198	30	3.20	-21.08	7.85	*Sus*	*mandibula*
	GrM-22776	EDAN0032	98BRUV018943	collagen	33	2	6019	29	3.12	-21.54	4.34	*Sus*	*mandibula*
	GrM-26625	EDAN0244	98BRUV015756	collagen	32	2	6080	27	3.30	-21.03	5.30	*Sus*	*scapula*
	GrM-26624	EDAN0243	98BRUV015993	collagen	20	2	6047	27	3.30	-22.11	5.00	*Sus*	*ulna*
	GrM-25038	EDAN0009	98BRUV002856	collagen	10	3	5530	30	3.20	-22.12	6.63	*Bos*	*tibia*
	GrM-22775	EDAN0006	98BRUV013556	collagen	15	3	5514	29	3.22	-22.57	7.00	*Bos*	*metacarpus*
	GrM-22781	EDAN0077	98BRUV013569	collagen	15	3	5569	27	3.28	-21.56	5.18	*Sus*	*mandibula*
	GrM-23702	EDAN0091	98BRUV015401	collagen	15	3	5500	45	3.30	-21.35	5.40	*Sus*	*mandibula*

The guidelines for the collagen extraction protocol (modified Longin et al [46]) are outlined in Dee et al. [47]. In brief, the bones were crushed into coarse pieces and decalcified using a mild acid (HCl_[aq]_, 4% w/vol, RT, 4 applications over 24h). Afterwards, they were rinsed with demineralized water (hereafter ‘DW’) to neutrality and subsequently subjected to base (NaOH_[aq]_, 1% w/vol, 30 min, RT) to eliminate humic acids. Neutralization with DW was then repeated, followed by another exposure to acid (HCl_[aq]_, 4% w/vol, 15 min, RT), and neutralization with DW. Afterwards, the samples were exposed to acidified DW (pH 3) and placed in an oven (80°C, 18 h). The next day the samples were filtered and air-dried to a crystalline solid. Aliquots of approximately 5.5 mg were weighed into Sn_[s]_ capsules and combusted in an Elemental Analyser (EA, Elemental Vario Isotope Cube) coupled to an Isotope Ratio Mass Spectrometer (IRMS, IsoPrime 100) and an automated cryogenic system which trapped the CO_2[g]_ into sealable glass vessels. This allowed for the determination of δ^13^C and δ^15^N values. The CO_2[g]_ was then graphitized over a Fe_[s]_ catalyst in a stoichiometric excess of H_2 [g]_. Finally, the graphite was pressed into Al_[S]_ cathodes and transferred to the MICADAS mass spectrometer (Ionplus AG, 200kV) for radioisotope analysis.

### Bayesian modelling and model specifications

For this study, we used the OxCal program [41]. In OxCal, radiocarbon dates are often arranged according to *prior* information, which expresses their stratigraphic relationships, or other types of grouping. The statistical analysis relies on a Monte Carlo Markov Chain (MCMC) algorithm which randomly samples the probability densities associated with the radiocarbon results. This sampling is done sequentially; each random sample is selected before the next random sample (hence the “chain”). Each new selection is independent of its predecessor but constrained by the prior information. Overall, the process involves the prior beliefs being updated by combining them with the likelihoods (data) to generate the posterior beliefs [48–50].

In this case study, the prior information is mainly the stratigraphical relationships between the dates. For this, we follow the contextual assignment of the dates in the published excavation reports [20, 21] (compare Table 2 with Figs 2 and 3). We excluded all dates with the possibility of a freshwater reservoir effect because the modeling exercise on the legacy data from Hardinxveld showed no contribution of these dates to the models [14]. The preferred models included only samples with an unambiguous context allocation, and excluded those that could not be assigned to a single phase or came from high-energy environments (i.e. colluvium).

**Fig 4:**
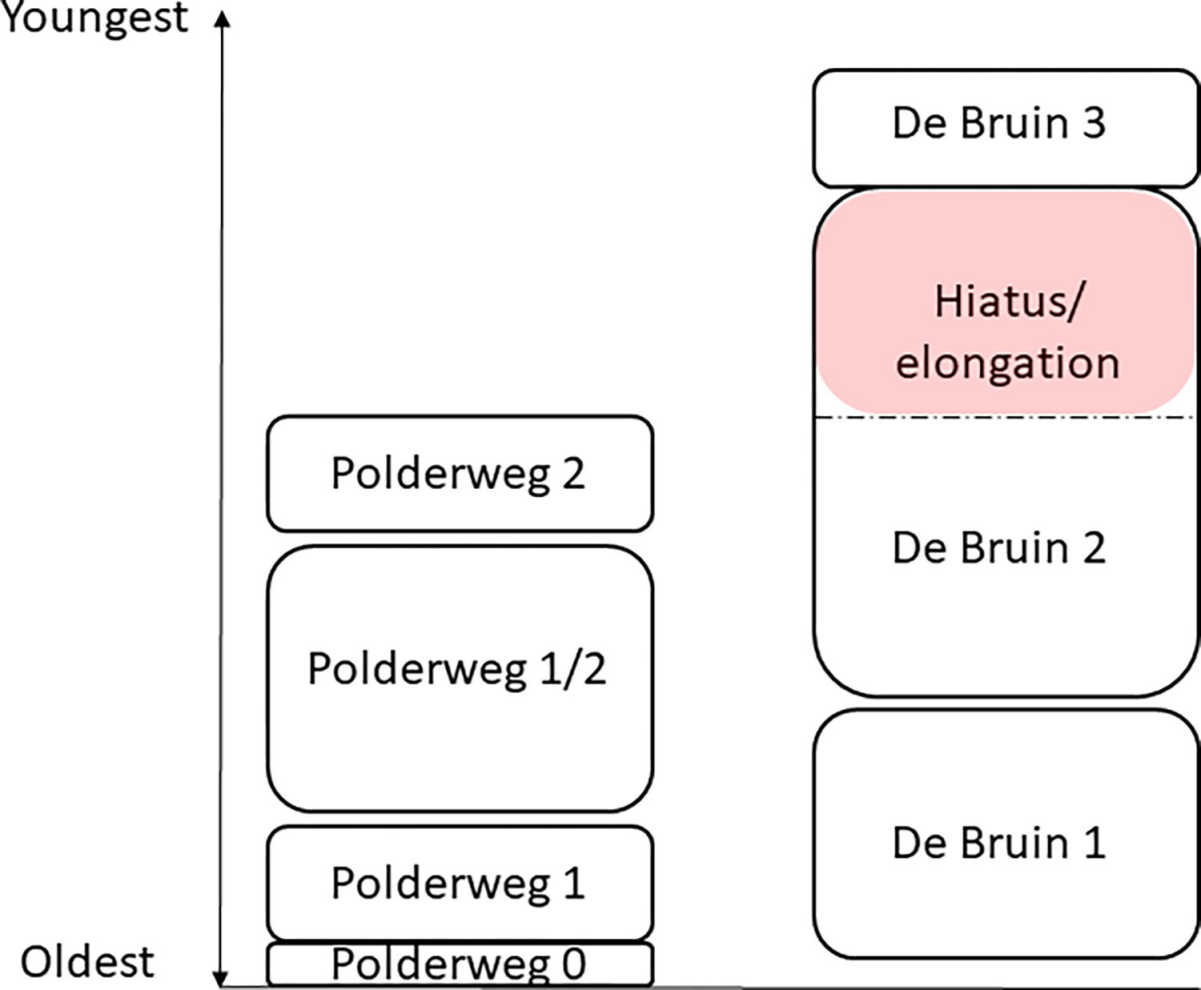
Scheme of occupation phases of Polderweg and de Bruin, as perceived in the models presented in this paper. For clarity, we refer to phases as ‘Polderweg 1’, ‘De Bruin 2’ etc. The elongation, marked in red, is caused by likely misplaced legacy dates, as explained below.

Both sites were modeled as a series of OxCal Phases enclosed within the command Sequence [41], with double Boundaries reflecting the archaeologically observed hiatuses separating the occupational phases (see Fig 4 for further information). An exception was made for the first two phases at Polderweg (Polderweg 0 and 1) because, according to the excavation reports, these phases are considered to follow each other and are even merged in stratigraphic interpretations in the excavation report [20, 21]. Phase 0 is, in essence, a single archaeological layer [37] deposited below the colluvium and ‘initial’ layers of phase 1 (see Fig 3). Hence, we placed only one Boundary between Polderweg 0 and 1 in the main model.

Radiocarbon dates placed inside OxCal Phases have no assumed order, which allows for the Bayesian process to approximate the most probable date ranges for each one [41]. We avoided enforcing order between dates because of unclear boundaries between layers within occupation phases, i.e. palimpsest effect [39], as discussed above.

We employed OxCal’s General Outlier model for the first-grade samples (with each outlier probability of 0.05) and the Charcoal Plus model for samples prone to the old wood effect (with each outlier probability set to 1.00 [51]). These outlier attributions act to downweigh the influence of any stratigraphically incongruent dates. For example, if a sample is considered an outlier by the model, it will have a minimal impact on the final result. However, the presence of such an outlier can also inform us of possible context missasociation or erroneous dating in the most unbiased and transparent way possible.

We used OxCal’s Date function as a convenient way of depicting the estimated time ranges of Phases as probability densities in absolute time [52, 53]. We also used Interval, Sum and Span (for code, see S.2.in S1 File) for the estimation of the duration of Phases.

To test the sensitivity of our main models, alternatives were prepared (see supplementary information) in which Outlier Analysis was not employed. Here, we considered the Overall Agreement of the models as an indication of the integrity of the proposed stratigraphical relationships. In such models, outliers were incrementally removed until the model reached >60% Overall Agreement. Note that the Agreement Index is not a wholly reliable measure of model congruency when Outlier Analysis is employed [54]

We also prepared models that employed an alternative contextual allocation of several macroremains. In these cases, there was strong circumstantial evidence of contextual missasociation that could not be identified by OxCal due to the lack of constraints between De Bruin 2 and 3 [Fig 8]. In addition, we ran a model which included the legacy dates potentially affected by reservoir effects (third grade), assigning them the After command (see code S.4.3., Fig S.5. in S1 File). The latter model produced comparable results to the main model, by downweighing the influence of third-grade samples where the offset was apparent.

## Results and discussion

### Radiocarbon dating

We followed ubiquitos criteria for collagen quality indicators, such as C:N atomic ratio [55] between 2.9 and 3.6, and a collagen yield of >0.5% (collagen content of the bone sample) [47]. The success rate was 90% of the total pretreated samples, with three failures out of 30 pretreatments.

**Fig 5:**
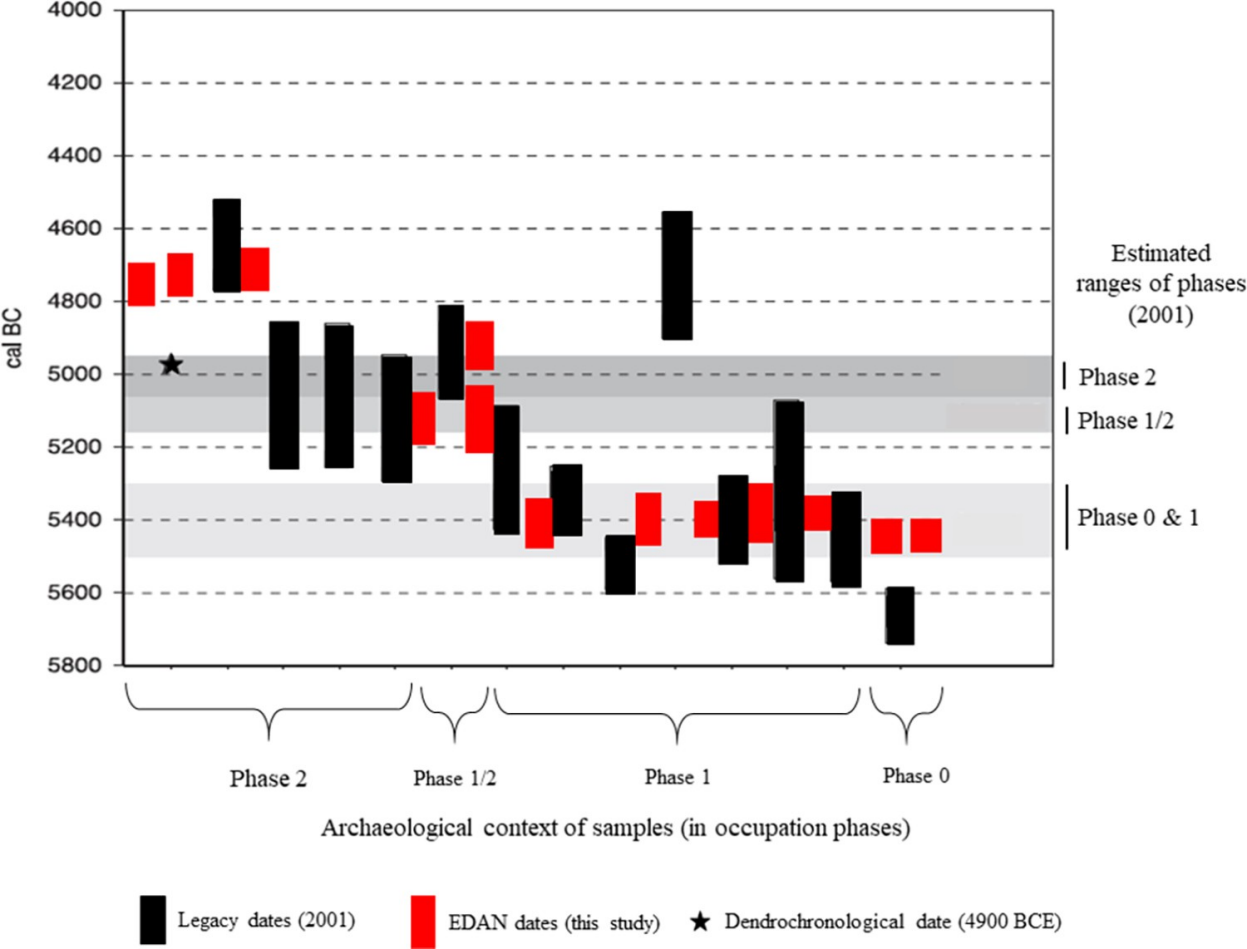
Comparison of newly acquired and legacy radiocarbon dates from Polderweg, expressed as 95% probability ranges. All dates are calibrated but not modelled. Notice the scattering of legacy dates as opposed to the tight clustering of new dates. New dates from phase 2 are significantly younger than the previously assumed time range of this phase. Figure adapted from Louwe Kooijmans & Mol 2001. [20] Fig 3.7. p. 69.

**Fig 6:**
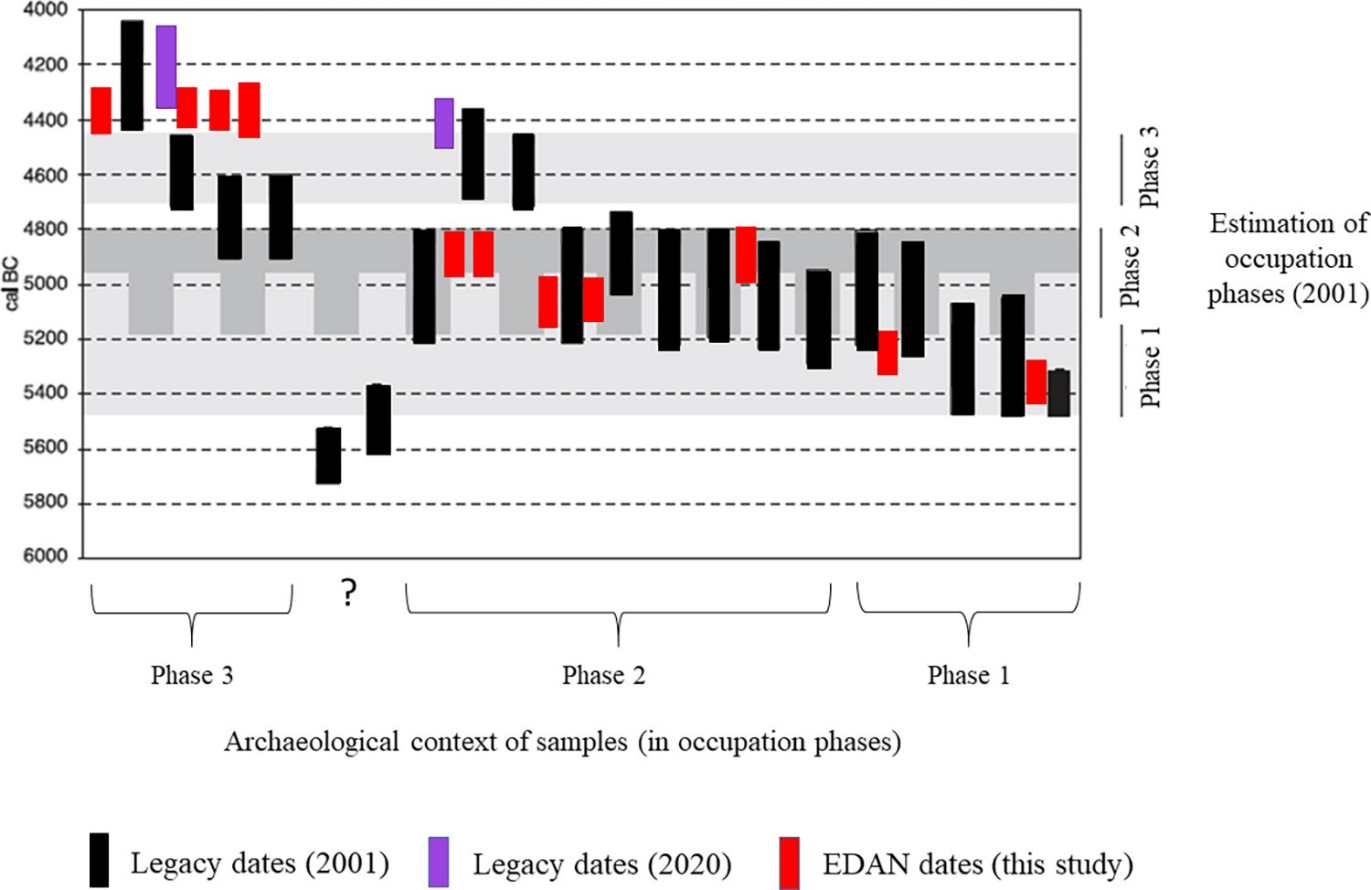
Comparison of newly acquired (excluding the two outliers, GrM-23514and GrM-23703, as explained above) and legacy radiocarbon dates from De Bruin, in 95% ranges. All dates are calibrated but not modelled. Notice the scattering of legacy dates as opposed to tight clustering of new dates. New dates from phase 3 are significantly younger than the previously assumed time range of this phase. Figure adapted from Mol & Louwe Kooijmans 2001. [21] Fig 35. p. 69.

In general, the new dates (Table 2) have substantially higher precision than the legacy dates and, consequently, tighter time ranges. Furthermore, the new dates appear to be clustered into groups that are in excellent agreement with each other and their contextual assignment into occupational phases. They do, however, exhibit an appreciable offset from the legacy data in the last occupational phases at both sites. The 2001 results ([16, 17], see also see Table 1 in S1 File) cluster to older ages (see Figs 5 and 6). We believe the discrepancy between the 2001 dates and the 2020 and new dates on both sites are largely due to reservoir effects and context missasociation. In our opinion, these sources of dating inaccuracy have affected the chronological interpretations in previous publications [14, 20–22].

Three new radiocarbon dates are outliers: GrM-23703, GrM-23514 and, unsurprisingly, GrM-22782 (*Lutra lutra*). Sample GrM-22782 yielded a result that was approximately 800 years older than the archaeological context which was expected due to the obvious aquatic diet common for otter species, further confirmed by stable isotope results (see Brusgaard et al. [15], Table 2).

However, samples GrM-23703 and GrM-23514 returned dates that are stratigraphically incongruent. GrM-23703 falls into the time ranges of De Bruin’s first occupational phase rather than the last. Any reservoir effect was ruled out due to the δ^13^C and δ^15^N reflecting a herbivorous diet ([15], see Table 2). According to the excavation report, the context of the respective feature had an ambiguous chronological attribution [40].

GrM-23514 returned a date that is 1000 years too young for its context (phase 1) and 500 years younger than the last occupation at Polderweg, ruling out the context missasociation within the site or any form of inbuilt age. The date actually coincides with De Bruin 3. However, Polderweg is assumed to have been submerged by this time [20, 35] and no other dated material provided comparable results. Both GrM-23514 and GrM-23703 generated very low collagen yields (~0.5%) [47]. Due to poor collagen preservation, the unusually young dates could have been a result of contamination. Repeated collagen extraction for both samples yielded no additional results. In conclusion, these two dates have to be considered with caution.

### Bayesian models

We combined the legacy and new dates from Polderweg and de Bruin in the following models to establish highly precise chronostratigraphies of these sites [see the schematic of the models, Fig 4]. The sensitivity models, wherein the impact of different approaches was tested, are listed in the supplementary information. Overall, both the Outlier Analysis and Agreement Index models produced comparable results.

#### Polderweg

**Fig 7:**
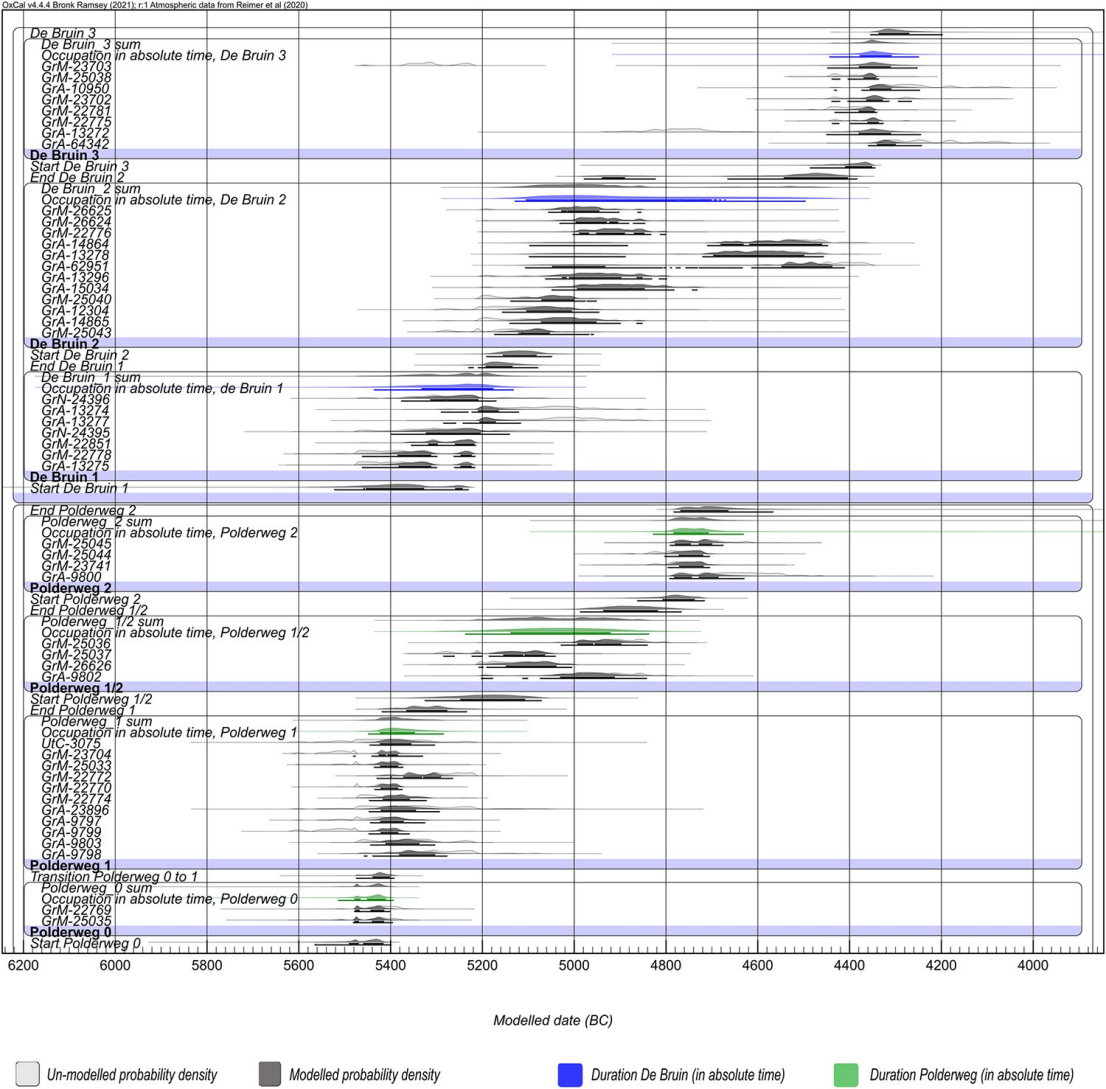
Plot with probability densities of modelled dates; Polderweg and De Bruin. Light gray presents unmodelled while dark gray show modelled probability densities. Estimated duration of phases are calculated under Date function, in green (Polderweg) and blue (De Bruin).

**Fig 8:**
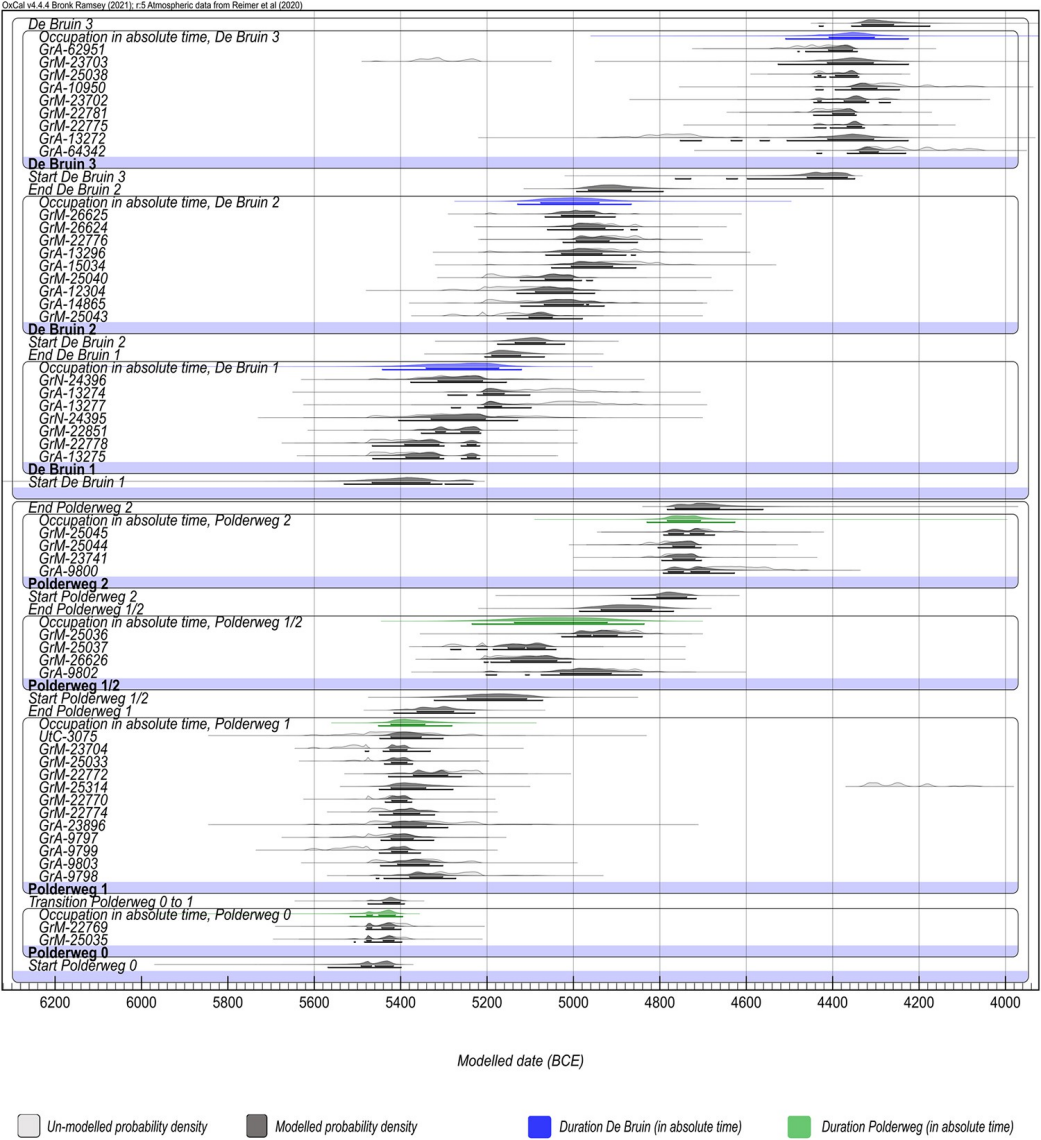
Plot with probability densities of modelled dates without intrusive dates; Polderweg and De Bruin. Light gray presents unmodelled while dark gray show modelled probability densities. Estimated duration of phases are calculated under Date function, in green (Polderweg) and blue (De Bruin).

Within the modeled occupation phases, the new dates cluster exceptionally well but exhibit an offset with respect to the phasing based on the legacy data in phases 1/2 and 2 [20, 22], contexts crucial to the narrative on the appearance of Swifterbant pottery [56, 57] (Figs 7 and 8). Phase 1/2 seems to extend over a longer time frame while Phase 2 shifts 200 years younger (Table 3, Fig 7). Previously, possibly erroneous legacy dates and a single dendrochronological date from, an allegedly intentionally, felled tree were taken as an anchor for dating Polderweg 2. This was problematic for several reasons. The tree was associated with this phase and human activity without certainty and its ambiguity was expressed in the excavation report ([20] p. 67). Our new dates confirm the discrepancy between said dendrochronological date and the remains of anthropogenic activity from this phase. Secondly, out of four legacy dates from this phase, only one was obtained on optimal material while the remainder were generated from charred food crust and the loose human cranium. This first grade legacy date (GrA-9800) appears to be in agreement with the new dates on herbivores.

**Table 3:** Comparison of the chronological interpretation of time ranges of occupational phases at Polderweg, in relation to the availability of reliable dates. Published ‘eye-balling ranges’ (2001), result of OxCal model (2007, functions & code unknown) and estimated with Date function in OxCal (this paper). The ranges given below represent the approximate intervals of time these occupations took place, as determined by the corresponding studies.

Phase	Louwe Kooijmans & Mol 2001	Mol & Van Zijverden 2007	This paper
Polderweg 0	5500 BCE	/	5500–5400 BCE
Polderweg 1	5500–5300 BCE	5400–5350 BCE	5450–5300 BCE
Polderweg 1/2	5300–5100 BCE	/	5200–4800 BCE
Polderweg 2	5100–4800 BCE	5200–5050 BCE	4800–4650 BCE
Number of reliable dates	8	8	21
Number of unreliable dates	7	7	1

Besides the alteration to the time range of the last phases, with new dates we were able to generate the first reliable absolute dates for Polderweg 0, or the first find layer, 37. There is only one legacy date from this period, sampled from a human burial associated with the contemporaneous remains of a dwelling structure. Due to its large offset from the rest of the legacy dates, it was discarded as erroneous (freshwater reservoir effect) and the timing of this phase was assumed to be just before the start of phase 1. The new dates confirm this interpretation, and indicate a negligible difference between probability densities of dates from phases 0 and 1 (Fig 7). Indeed, the sensitivity tests without Outlier Analysis, reached a significantly higher Agreement (> 60%) when Polderweg 0 and 1 were merged into a single Phase (Code S.4.6.).

#### De Bruin

The plot of modeled probability densities for De Bruin, divided into the three main occupational phases separated by hiatuses, is depicted in Figs 7 and 8. Besides the previously mentioned GrM-23703, potentially untrustworthy due to low collagen preservation, the rest of the new dates are in excellent agreement with their allocated occupation phase.

Phase 2 is unusually elongated due to the way the dates form two clusters with the younger cluster extending over an observed hiatus between De Bruin 2 and 3 (Fig 7, Code S.2 in S1 File). The older cluster comprises four first-grade legacy dates and all (five) newly dated samples, while the younger cluster features three first-grade legacy dates (GrA-14864, GrA-13278, GrA-62951). Our suspicion over the integrity of the latter has been addressed elsewhere [14]. In short, date GrA-62951 was sampled from the remains of a goat/sheep which was deemed to be misplaced and likely to be from the context of De Bruin 3, as stated in the excavation report [18]. This was further confirmed by our study because this date is in excellent agreement with the new dates from De Bruin 3, layer 15 (GrM-22775, GrM-22781 and GrM-23702), which is the lowest layer of phase 3, deposited on top of the hiatus. Since both archaeological and radiocarbon evidence suggests context missasociation, we believe this to be good evidence for displacement.

The other two in the suspicious cluster of legacy dates (GrA-13278 and GrA-14864) are short-lived dates on macroremains, which were allocated to the end of De Bruin 2. However, sample GrA-13278 (charred *Alnus* seed) comes from layer 32 which extends across both phases 2 and 3 [29]. Hence, we cannot be sure that this sample is not associated with a sedimentation hiatus on top of De Bruin 2 or with phase 3. Lastly, GrA-14864, associated with layer 20, was collected as a peat bulk sample (monolith tin) with uncharred *Corylus* shell fragments [29]. There is no evidence that this sample was associated with an anthropogenic activity. Indeed, in Mol and Zijverden 2007 [22], GrA-14864 and GrA-13278 were interpreted as *terminus ante quem* for phase 2, collected from the top of the phase as presumably non-archeological samples. That would make them contextually equally associable with the hiatus. However, because there is no constraint after De Bruin 2, the results of the Bayesian models continue to elongate the probability distribution of this phase, without detecting these dates as outliers.

The context ambiguity here is further confirmed by date GrA-13272, from the beginning of De Bruin 3, which returned ranges that overlap with De Bruin 2, despite the sizeable hiatus layer separating these phases. This could have been due to erosion in layer 20 [21], a mechanism which introduces older remains into the younger context.

To investigate this issue, we dated the remains of herbivores from the same layers as the suspicious dates. All (GrM-22775, GrM-26624, GrM-26625) returned older ages, in excellent agreement with the older cluster. This corresponds well with the archaeological interpretation of a long hiatus in human occupation following De Bruin 2. If we modelled this site with entirely new dates, there would be no elongation of phase 2 (See code S.4.5. in S1 File).

In our sensitivity tests models without Outlier Analysis, the sheep/goat bone GrA-62951 and the macroremain sample GrA-13272 had to be manually removed for the model to reach above 60% Agreement. (See code S.4.2. in S1 File). This serves as an additional argument towards these samples being intrusive. It is, however, important to note that the model with Outlier Analysis leaves a small probability that phase 2 does stretch as far as phase 3. Further evidence is required to discount this possibility. Indeed, we conducted simulations to see how many more dates this would require, and at least 17 from end phase 2, consistent with the older cluster, would be required to statistically demonstrate that the younger cluster is erroneous (See code S.4.3. in S1 File). Because we already had solid evidence of context missasociation for these samples, we decided that this exercise was not cost-effective.

In the comparison Model 2 (Fig 8, code S.3. in S1 File), where the presumably intrusive dates that could not be detected by OxCal were excluded, the estimated time ranges of De Bruin 2 extend between 100 and 200 years younger than previous interpretations (2001 [21]; 2007 [22]; see Table 4). This is the context where Swifterbant pottery appears at De Bruin [58] which is now contemporaneous with Polderweg 1/2 and precedes Polderweg 2. This would shift the first appearance of pottery from Polderweg 2 to De Bruin 2.

**Table 4:** Comparison of chronological interpretation of the duration of occupation phases at De Bruin, in relation to the number of reliable dates. Published ‘eye-balling ranges’ (2001), the result of OxCal model (2007, function & code unknown) and estimated with Date function in OxCal (this paper). The ranges given below represent the approximate intervals of time these occupations took place, as determined by the corresponding studies.

Phase	Mol & Louwe Kooijmans 2001	Mol & Van Zijverden 2007	This paper
De Bruin 1	5500–5300 BCE	5200–5100 BCE	5450–5150 BCE
De Bruin 2	5300–5000 BCE	5040–4940 BCE	5100–4800 BCE
De Bruin 3	4700–4500 BCE	4550–4500 BCE	4450–4250 BCE
Number of reliable dates	12	12	23
Number of unreliable dates	8	8	1

The most noticeable difference is the approximated time range of De Bruin 3 (4450–4250 BCE), which was initially considered to have been covered with alluvial clay by 4500 BCE due to rising groundwater levels and peatmarsh extension [21]. As previously discussed, this is not reliable and could have been influenced by local factors, such as beaver activity [31–33]. Although wet for most of the year, localized conditions may have been such that short-lived ‘camping’ was still possible during the dry season (e.g. this has been observed at the early fifth-millennium site of Almere-Hoge Vaart [59]). Only, in the case of full peat marsh conditions with permanent peat accumulation, any form of dwelling may have become impossible or undesirable. As there is erosive contact between this top clay layer and the underlying layers with the archaeological remains, there must exist a hiatus [21]. Thus, the chronology presented in this study introduces new information, important for the understanding of the landscape development at Hardinxveld sites.

#### The contemporaneity of De Bruin and Polderweg and the earliest presence of domesticated animals in the Swifterbant culture

There has been significant discussion over the relationship between these sites, with scholars generally adopting the idea that they were most likely shared by the same, or cooperating, communities [7, 17, 26]. Depending on the time period and other factors such as, for example, the availability of dry soil, the functions of the sites might have alternated with respect to each other [17, 26, 31, 33].

**Fig 9:**
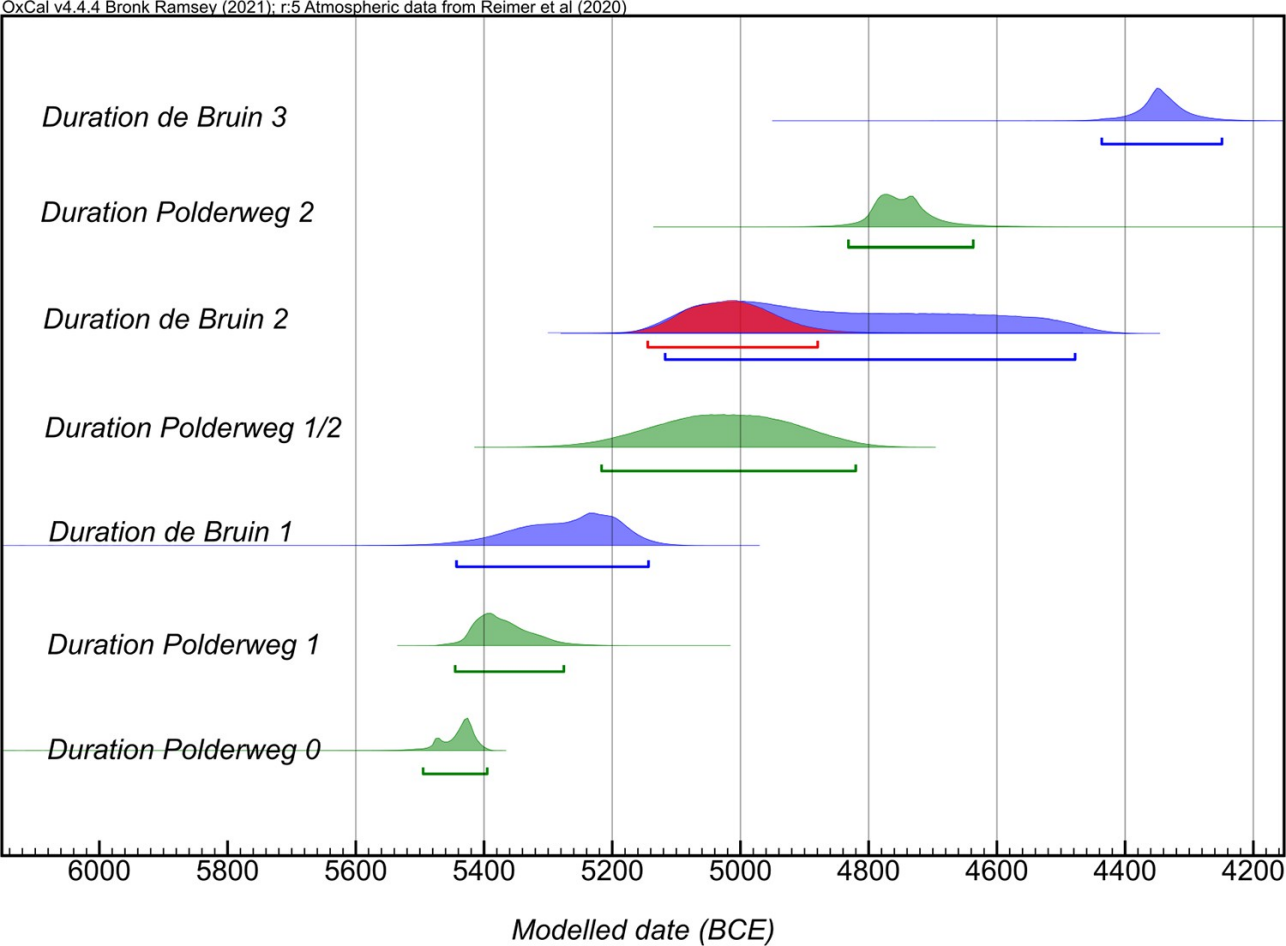
Date ranges of occupation phases of Polderweg and De Bruin in absolute time, estimated with Date function. Probability distribution in red marks a phase modelled without the intrusive legacy dates from the end De Bruin 2. Note that the probability of phase 2 gravitates towards older ages.

To try to understand their chronological relationship, we compared the time ranges of human activity at these two sites, based on our models. Fig 9 illustrates how these sites overlapped or preceded each other in terms of occupational phasing. According to the results, the first small-scale human occupation began in Polderweg 0, most likely before the beginning of human activity at De Bruin. Shortly after, there is an upsurge in the evidence of the human presence in Polderweg 1 which was (partly) contemporaneous with De Bruin 1.

Similarly, Polderweg 1/2 and De Bruin 2 were likely contemporaneous. This is the time when there seems to have been an increase in the occupation intensity at De Bruin 2 while in Polderweg 1/2 the evidence of human presence decreases [20, 21]. As mentioned before, with De Bruin 2 preceding Polderweg 2, the oldest Swifterbant pottery [56–58] seems to have emerged at De Bruin rather than Polderweg, between 5100 and 4800 BCE. Indeed, scarce evidence of pottery sherds at Polderweg ½ [57], now contemporaneous with the emergence of pottery at De Bruin 2, may serve as an additional argument for the relationship between these two sites.

The following phase, Polderweg 2 is supported by little archaeological evidence, as discussed above. Hence, besides the site abandonment at around 4600 BCE, there is too little data to conclude anything more on the nature of this occupation phase. De Bruin shows no habitation during this time.

**Fig 10:**
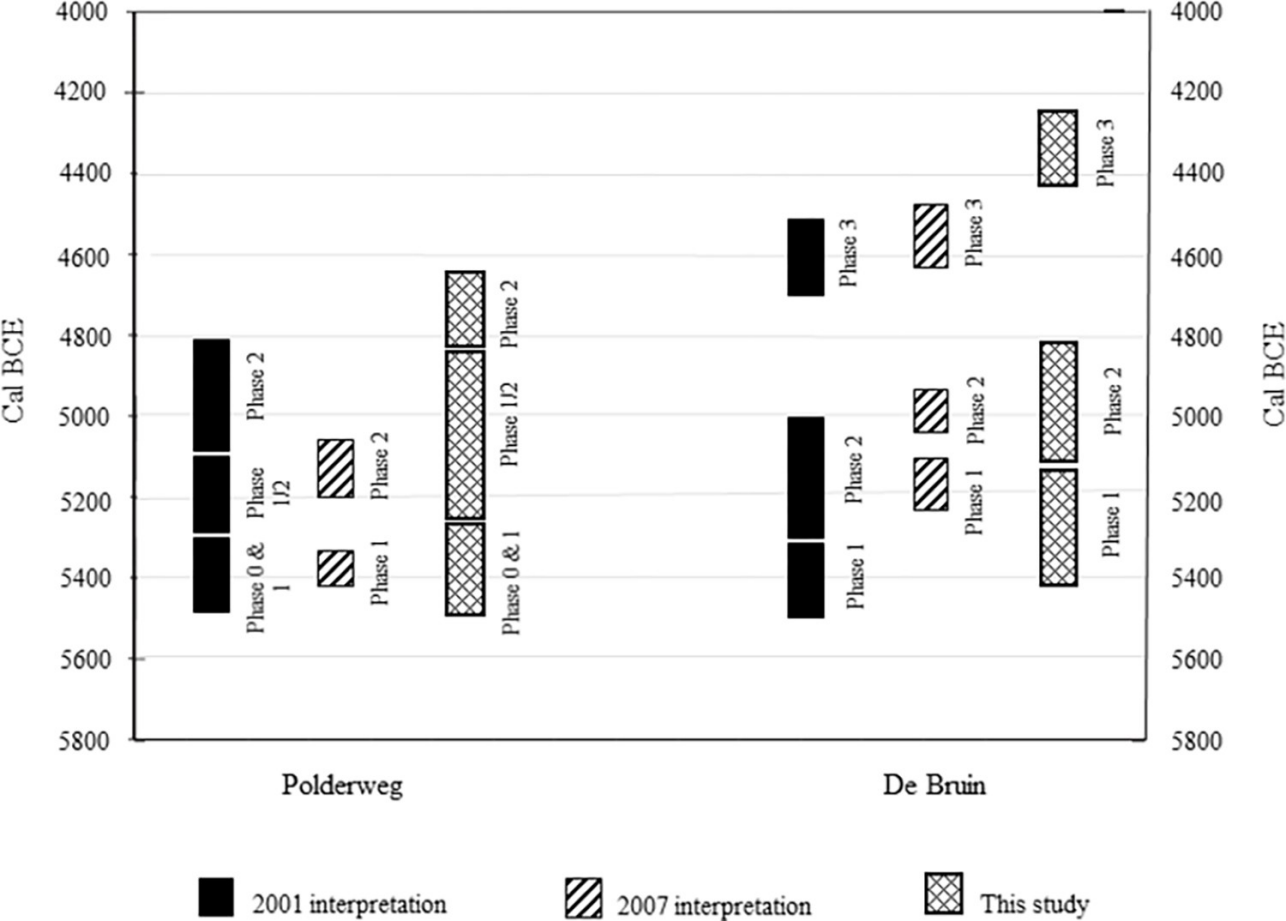
Visual comparison of interpretations of time ranges of occupation phases at Hardinxveld sites. The most significant differences are phases 1/2 and 2 Polderweg and phases 2 and 3 De Bruin.

According to the newly acquired dates (Figs 8 and 10), the period of low activity lasted until 4450 BCE, as evidenced by the hiatus layer in the stratigraphic profiles of De Bruin [21]. Finally, De Bruin, as the highest and only dry dune in the area, served as a modest base camp between 4450 BCE and 4250 BCE (De Bruin 3). New dwelling structures were constructed on the top of the dune, this time featuring novelties such as a small number of domesticated sheep/goat, likely dairy production or consumption, and an appearance of significantly smaller specimens of *Sus* and *Bos*, albeit without signs of dietary or population management [15, 16, 18, 21]. During this time frame, the presence of sheep and goat, also in low numbers, has been noted at another Swifterbant site in Scheldt valley, Bazel ‘‘Sluis” [60]. There, the radiocarbon-dated younger cluster of sheep/goat appears to be in excellent agreement with De Bruin 3 and, interestingly, the older cluster (approximately 4700–4500 BCE) overlaps with a sizeable hiatus observed at De Bruin [Fig 2, 37, 60]. Similar to the Hardinxveld sites, there appears to be smaller-sized *Bos* at Bazel “Sluis” but no conclusive evidence has been brought forward of their domesticated status or dietary management [37]. In fact, the dominance of small-sized metapodial bones at Bazel ‘‘Sluis”, commonly used as tools, further indicates the possibility that the presence of domesticated animals at this time, within Swifterbant culture, was a product of exchange with farming communities, and likely predating animal husbandry [37]. This might have been a regional pattern.

In addition to this, De Bruin 3 is now chronologically closer to the direct dates on sheep/goat species from the nearby site of Brandwijk-Kerkhof (4250–3950 BCE, 95%) that also exhibits evidence of dairy production or consumption [13, 61, 62]. However, since the chronostratigraphies of both Brandwijk and Bazel “Sluis” are unresolved, it remains difficult to conduct a more detailed chronological comparison between these sites.

## Conclusion

We conducted a high-resolution Bayesian chronological investigation of the twin sites of Hardinxveld Giessendam Polderweg and De Bruin, which are the best-preserved Late Mesolithic sites in the Dutch wetlands. Because of the presence of bones from domestic animals in the last occupational phase of De Bruin, these sites are critical for the study of early animal husbandry. The existence of domesticated sheep/goat as well as possible dairy production, indicate a certain shift in the approach to food sources. With the appearance of significantly smaller *Sus* and *Bos*, albeit without any evidence of animal husbandry, these sites reflect an interesting regional pattern common elsewhere in Europe and are, thus, crucial for the establishment of the chronological framework within the debate on early animal husbandry in northwestern Europe. To rectify the limitations intrinsic to the legacy radiocarbon dataset, we obtained radiocarbon dates on collagen samples from one aquatic and 26 from selected herbivorous mammals with clear context associations. Following a re-evaluation of the legacy data, we chose reliable legacy dates and combined them with the new dates in Bayesian models which also allowed us to explore the chronological relationship between these two sites. Polderweg and De Bruin seem to have had a largely overlapping and partly alternating chronological pattern, with the introduction of pottery now associated with De Bruin 2, rather than Polderweg 2, as is commonly assumed. In addition to this, the possible evidence of dairy fats in one of the pots at De Bruin 3 (4450–4250 BCE) seems to be closer in time to similar evidence from the nearby site of Brandwijk (4250–3900 BCE). The results of this analysis have a substantial impact on the interpretation of the appearance of domesticated animals in Dutch wetlands which are now associated with a carefully dated context that is approximately 200 years younger than previously estimated. This makes the appearance of domesticates at Hardinxveld significantly younger than the oldest evidence in Scheldt valley (4700–4500 BCE). In fact, the two oldest sheep/goat remains from modern day Belgium seem to overlap with the hiatus period at Hardinxveld sites while the remaining Bazel‘‘Sluis” dates fit well with the last phase at De Bruin.

Ultimately, we were able to establish a credible chronology of the initial emergence of domesticated animals in the Dutch wetlands and explore an interesting chronological relationship with the oldest such evidence of the Swifterbant culture in Belgium. The results of this study offer an invaluable baseline for the study of the early Neolithization process in the Netherlands and beyond.

## Supporting information

S1 File (DOCX)
