# Supplementary material for: High-resolution Bayesian chronology of the earliest evidence of domesticated animals in the Dutch wetlands (Hardinxveld-Giessendam archaeological sites)
Source: PLoS One. 2023 Jan 24;18(1):e0280619. doi: 10.1371/journal.pone.0280619 (PMC9873193; doi:10.1371/journal.pone.0280619)
Supplement: S1 File — (DOCX) [file pone.0280619.s001.docx]

Supplementary material for:

**High**-**resolution Bayesian chronology of the earliest evidence of domesticated animals in the Dutch wetlands (Hardinxveld-Giessendam archaeological sites)**

Merita Dreshaj^1,2*^, Michael Dee^2^, Nathalie Brusgaard^1^, Daan Raemaekers^1^ & Hans Peeters^1^

^a^ Groningen Institute of Archaeology, University of Groningen, Groningen, Netherlands

^b^ Centre for Isotope Research, Energy Academy, Groningen, Netherlands

S.1 Table. List of legacy radiocarbon data from Hardinxveld-Giessendam sites

S.2 Code for Main model 1 (reference to Fig 7)

S.3 Code for Main model 2 (reference to Fig 8)

S.4. Codes for sensitivity test models

S.4.1. Main model 1, no Outlier model

S.4.2. Main model 1, no Outlier model, excluded outliers (> 60% Overall Agreement)

S.4.3. Main model 1 with third grade legacy dates (command After)

S.4.4 Model De Bruin with simulations (with Outlier model)

S.4.5 Model with solely new (EDAN) dates, no Outlier Analysis

S.4.6. Polderweg, Model without Outlier Analysis, merged Phase 0 and 1

S.5. Plot with modelled probability densities of Hardinxveld sites, with Outlier Analysis; included third grade legacy dates

S.1 Table. List of legacy radiocarbon data from Hardinxveld-Giessendam sites

Table 1: Legacy radiocarbon data from Hardinxveld Giessendam sites

| Archaeological site | Lab. No. | Site sample ID | Sample material | Sample type | Context (layer, feature) | Date (cal. BP) | Uncertainty | δ ^13^C (‰) | δ ^15^N (‰) | | Reference | Grade | Reason Rejected | Remark |
| --- | --- | --- | --- | --- | --- | --- | --- | --- | --- | --- | --- | --- | --- | --- |
| Polderweg | GrA-9800 | 18.1.1. | Macroremains (in peat bulk) | Unknown species | Top unit 6, layer 10 or 11, peat | 5780 | 50 | -28.14 |  | | Louwe Kooijmans & Mol 2001; CIO submission forms | 1 |  | Peat bulk, uncharred |
|  | GrA-11829 | vnr.3026 | Foodcrust | Charred remains on pottery | Unit 6, layer 3 | 6130 | 50 | -29.33 |  | | Louwe Kooijmans & Mol 2001; CIO submission forms | 3 | Reservoir effect |  |
|  | GrA-11841 | vnr.3288 | Foodcrust | Charred remains on pottery | Unit 6, layer 20 | 6140 | 50 | -28.08 |  | | Louwe Kooijmans & Mol 2001; CIO submission forms | 3 | Reservoir effect |  |
|  | GrA-11830 | vnr.24038 | bone | Human, cranium | Unit 6, layer 3 | 6170 | 50 | -24.32 | 16.5 | | Louwe Kooijmans & Mol 2001; CIO submission forms | 3 | Reservoir effect |  |
|  | GrA-9802 | 11/783 | macroremains | fruit cone (*Alnus*) | Unit 6, layer 12, peat | 6050 | 50 | -27.07 |  | | Louwe Kooijmans & Mol 2001; CIO submission forms | 1 |  | uncharred |
|  | GrA-9798 | 11/818 | macroremains | seeds (*Cornus*) | The base of unit 6, layer 26, peat | 6320 | 50 | -25.86 |  | | Louwe Kooijmans & Mol 2001; CIO submission forms | 1 |  | uncharred |
|  | GrA-9803 | 11/864 | macroremains | Seeds (*Iris, Scirpus*) | Top of unit 4, layer 29, peat | 6380 | 50 | -24.03 |  | | Louwe Kooijmans & Mol 2001; CIO submission forms | 1 |  | uncharred |
|  | GrA-9799 | 11/919 | macroremains | Acorn (*Quercus*) | Base of unit 4, layer 29, next to the previous sample, peat | 6540 | 50 | -25.05 |  | | Louwe Kooijmans & Mol 2001; CIO submission forms | 1 |  | uncharred |
|  | GrA-10902 | vnr. 8350 | bone | *Canis canis* | Unit 3, layer 30 or 40, grave 4 | 5880 | 60 | -23.19 |  | | Louwe Kooijmans & Mol 2001; CIO submission forms | 3 | Reservoir effect |  |
|  | GrA-9807 | 26/1007 | bone | *Canis Canis* | Unit 3, layer 40 or 30, grave 3 | 6650 | 50 | -21.03 |  | | Louwe Kooijmans & Mol 2001; CIO submission forms | 3 | Reservoir effect |  |
|  | GrA-9797 | 25/1003 | macroremains | charcoal | Base of the unit 3, layer 40, pit 5 (fill) | 6480 | 50 | -25.1 |  | | Louwe Kooijmans & Mol 2001; CIO submission forms | 2 |  |  |
|  | GrA-23896 | 25/1003 | macroremains | charcoal | Base of a unit 3, layer 40, next to the previous sample, pit 5 (fill) | 6390 | 100 | -25.54 |  | | Louwe Kooijmans & Mol 2001; CIO submission forms | 2 |  |  |
|  | GrA-9804 | 25/1004 | bone | human | Unit 1, layer 60, grave 1 | 6820 | 50 | -22.6 |  | | Louwe Kooijmans & Mol 2001; CIO submission forms | 3 | Reservoir effect |  |
|  | UtC-3075 | AAO | macroremains | charcoal | Find layer 1 | 6450 | 90 |  |  | | Louwe Kooijmans & Mol 2001; CIO submission forms | 1 |  |  |
| Hardinxveld Giessendam de Bruin | GrA-10950 | vnr. 13254 | macroremains | Charred seeds of fruits of *Galium*, hazelnut and *Urtica diotica* (nettle) | Layer 10, pit 21, west section | 5430 | 60 | -25.37 |  | Mol & Louwe Kooijmans 2001; CIO submission forms | | 1 |  |  |
|  | GrA-13320 | vnr.20.692 | Food crust | Charred remains on pottery (similar to Brandwijk and Hazendonk style) | Layer 10, pit 22, end of phase 3 | 5730 | 50 | -29.28 |  | Mol & Louwe Kooijmans 2001; CIO submission forms | | 3 | Reservoir effect |  |
|  | GrA-64342 | 98BRUV016824 | Bone | *Ovis ammon f. aries,* metatarsus | Layer 15 | 5380 | 40 | -22.5 | 6.60 | CҪakirlar et al. 2020 | | 1 |  |  |
|  | GrA-13272 | vnr.13.249 | macroremains | Charred seeds of *Tilia* | Layer 15, pit 16, base od phase 2B | 5900 | 50 | -28.62 |  | Mol & Louwe Kooijmans 2001; CIO submission forms | | 1 |  | Same pit (16) as sample GrA-13278 |
|  | GrA-13317 | 20.694 | Food crust | Charred remains on pottery | Pit 13, feature 1004 (posthole, most likely a house), burried in layer 17 | 5880 | 50 | -27.7 |  | Mol & Louwe Kooijmans 2001; CIO submission forms | | 3 | Reservoir effect |  |
|  | GrA-11816 | 20.686 | bone | Human, left humerus | Feature 1021, pit 21, burial G1 | 6710 | 50 | -20,86 |  | Mol & Louwe Kooijmans 2001; CIO submission forms | | 3 | Reservoir effect, unknown context |  |
|  | GrA-11815 | vnr. 20.685 | bone | Human, left humerus | Feature 1004, pit 22, burial G2 | 6530 | 50 | -21,65 |  | Mol & Louwe Kooijmans 2001; CIO submission forms | | 3 | Reservoir effect, unknown context |  |
|  | GrA-13315 | vnr. 20.695 | Food crust | Charred remains on pottery | Feature 1001, pit 18, layer 30, base phase 2B | 6070 | 50 | -28,17 |  | Mol & Louwe Kooijmans 2001; CIO submission forms | | 3 | Reservoir effect |  |
|  | GrA-14864 | DB3 | macroremains | Peat bulk with Seeds of *Corylus* & 4 fragments of shells, pollen tray | layer 20, pit 24 | 5685 | 50 | / |  | Mol & Louwe Kooijmans 2001; CIO submission forms | | 1 |  | Uncharred seeds + peat bulk |
|  | GrA-13278 | vnr. 13.250 | macroremains | Charred seeds of *Alnus* fruit cones | Layer 32, pit 16, top of phase 2A | 5730 | 50 | -28,33 |  | Mol & Louwe Kooijmans 2001; CIO submission forms | | 1 |  | Same pit (16) as sample GrA-13272 |
|  | GrA-13313 | vnr. 20.696 | Food crust | Charcoal remains on (imported?) pottery, food crust | Layer 20, pit 15 | 6090 | 50 | -27,44 |  | Mol & Louwe Kooijmans 2001; CIO submission forms | | 3 | Reservoir effect |  |
|  | GrA-62951 | 98BRUV06258 | Bone | *Ovis ammon f. aries/Capra aegagrus f. hircus* | Layer 33 | 5610 | 40 | -22.9 | 4.80 | Çakirlar et al., 2020. | | 1 |  |  |
|  | GrA-15034 | DB4 | macroremains | Charred seeds of *Tilia* fruits, 1 whole and 2 fragmented | Layer 33, pit 24 | 6010 | 55 | / |  | Mol & Louwe Kooijmans 2001; CIO submission forms | | 1 |  |  |
|  | GrA-13318 | 12 vnr. 20.693 | Food crust | Charred remains on (imported?) pottery | Layer 40, pit 19 | 6100 | 50 | -27,12 |  | Mol & Louwe Kooijmans 2001; CIO submission forms | | 3 | Reservoir effect |  |
|  | GrA-13296 | vnr. 13.251 | macroremains | Charred seeds of *Corylus* (hazelnut) and Tilia nut | Layer 30, pit 16, lower half of phase 2A | 6050 | 50 | -26,52 |  | Mol & Louwe Kooijmans 2001; CIO submission forms | | 1 |  |  |
|  | GrA-14865 | DB5 | macroremains | Peat bulk containing *Corylus* seeds and shell fragments | Layer 135, pit 24 | 6120 | 50 | / |  | Mol & Louwe Kooijmans 2001; CIO submission forms | | 1 |  | Uncharred seeds + peat bulk; sampled from monolith tin |
|  | GrA-12304 | DB6 | macroremains | Bulk of peat & sand with seeds of *Tilia* fruit and segments of unspecified fruit, | Layer 135, pit 24 | 6170 | 50 | / |  | Mol & Louwe Kooijmans 2001; CIO submission forms | | 1 |  | Uncharred seeds + peat bulk, sampled from monolith tin |
|  | GrA-13277 | vnr. 13.252 | charcoal | / | Layer 36, pit 16, top of phase 1 | 6100 | 50 | -26,81 |  | Mol & Louwe Kooijmans 2001; CIO submission forms | | 2 |  |  |
|  | GrA-13274 | vnr. 20.679 | wood | Remains of a fish trap*, Cornus sanguinea* | Layer 35, pit 12, feature 1003 | 6130 | 50 | -27,13 |  | Mol & Louwe Kooijmans 2001, C.C.Bakels et al. 2001. | | 2 |  |  |
|  | GrN-24396 | vnr. 20.702 | wood | Canoe*, Tilia sp*. | Layer 35, pit 24, base phase 2A | 6310 | 60 | -26,71 |  | Mol & Louwe Kooijmans 2001, C.C.Bakels et al. 2001 | | 2 |  |  |
|  | GrN-24395 | vnr. 20.699 | wood | Canoe, *Tilia sp.* | The bottom part of layer 35, pit 8 | 6310 | 90 | -28,2 |  | Mol & Louwe Kooijmans 2001, C.C.Bakels et al. 2001 | | 2 |  |  |

S.2 Code for Main model 1 (reference to Fig 7)

Options()

{

Resolution=1;

};

Plot()

{

Outlier_Model("General",T(5),U(0,4),"t");

Outlier_Model("IA",Prior("Charcoal_Plus"),U(0,3),"t");

Sequence()

{

Boundary("Start Polderweg 0");

Phase("Polderweg 0")

{

R_Date("GrM-25035", 6492, 29)

{

Outlier("General", 0.05);

};

R_Date("GrM-22769", 6473, 29)

{

Outlier("General", 0.05);

};

Date("Occupation in absolute time, Polderweg 0")

{

color="green";

};

Interval("Polderweg 0, Interval");

Span("Polderweg 0");

Sum("Polderweg 0 sum")

{

};

};

Boundary("Transition Polderweg 0 to 1");

Phase("Polderweg 1")

{

R_Date("GrA-9798", 6320, 50)

{

Outlier("General",0.05);

};

R_Date("GrA-9803", 6380, 50)

{

Outlier("General",0.05);

};

R_Date("GrA-9799", 6540, 50)

{

Outlier("General",0.05);

};

R_Date("GrA-9797", 6480, 50)

{

Outlier("IA", 1);

};

R_Date("GrA-23896", 6390, 100)

{

Outlier("IA", 1);

};

R_Date("GrM-22774", 6420, 29)

{

Outlier("General", 0.05);

};

R_Date("GrM-22770", 6483, 22)

{

Outlier("General", 0.05);

};

R_Date("GrM-22772", 6298, 29)

{

Outlier("General", 0.05);

};

R_Date("GrM-25033", 6502, 30)

{

Outlier("General", 0.05);

};

R_Date("GrM-23704", 6550, 30)

{

Outlier("General", 0.05);

};

R_Date("UtC-3075", 6450, 90)

{

Outlier("IA", 1);

};

R_Date("GrM-23514", 5383, 26)

{

Outlier("General", 0.05);

};

Interval("Polderweg 1, Interval");

Sum("Polderweg 1 sum")

{

};

Span("Polderweg 1 span");

Date("Occupation in absolute time, Polderweg 1")

{

color="green";

};

};

Boundary("End Polderweg 1");

Boundary("Start Polderweg 1/2");

Phase("Polderweg 1/2")

{

R_Date("GrA-9802", 6050, 50)

{

Outlier("General",0.05);

};

R_Date("GrM-26626", 6167, 29)

{

Outlier("General", 0.05);

};

R_Date("GrM-25037", 6223, 29)

{

Outlier("General", 0.05);

};

R_Date("GrM-25036", 6017, 29)

{

Outlier("General", 0.05);

};

Date("Occupation in absolute time, Polderweg 1/2")

{

color="green";

};

Interval("Polderweg 1/2, Interval");

Sum("Polderweg 1/2 sum")

{

};

Span("Polderweg 1/2 span");

};

Boundary("End Polderweg 1/2");

Boundary("Start Polderweg 2");

Phase("Polderweg 2")

{

R_Date("GrA-9800", 5780, 50)

{

Outlier("General",0.05);

};

R_Date("GrM-23741", 5889, 27)

{

Outlier("General", 0.05);

};

R_Date("GrM-25044", 5905, 29)

{

Outlier("General", 0.05);

};

R_Date("GrM-25045", 5841, 29)

{

Outlier("General", 0.05);

};

Date("Occupation in absolute time, Polderweg 2")

{

color="green";

};

Interval("Polderweg 2, Interval");

Sum("Polderweg 2 sum")

{

};

Span("Polderweg 2 span");

};

Boundary("End Polderweg 2");

};

Sequence()

{

Boundary("Start de Bruin 1");

Phase("de Bruin 1")

{

R_Date("GrA-13275", 6420, 50)

{

Outlier("General", 0.05);

};

R_Date("GrM-22778", 6427, 53)

{

Outlier("General", 0.05);

};

R_Date("GrM-22851", 6319, 29)

{

Outlier("General", 0.05);

};

R_Date("GrN-24395", 6310, 90)

{

Outlier("IA", 1);

};

R_Date("GrA-13277", 6100, 50)

{

Outlier("IA", 1);

};

R_Date("GrA-13274", 6130, 50)

{

Outlier("IA", 1);

};

R_Date("GrN-24396", 6310, 60)

{

Outlier("IA", 1);

};

R_Date("GrM-23703", 6345, 28)

{

Outlier("General", 0.05);

};

Span("De Bruin 1 span");

Sum("De Bruin 1 sum")

{

};

Date("Occupation in absolute time, de Bruin 1")

{

color="blue";

};

Interval("De Bruin 1, Interval");

};

Boundary("End de Bruin 1");

Boundary("Start de Bruin 2");

Phase("de Bruin 2")

{

R_Date("GrM-25043", 6213, 26)

{

Outlier("General", 0.05);

};

R_Date("GrA-14865", 6120, 50)

{

Outlier("General", 0.05);

};

R_Date("GrA-12304", 6170, 50)

{

Outlier("General", 0.05);

};

R_Date("GrM-25040", 6138, 26)

{

Outlier("General", 0.05);

};

R_Date("GrA-15034", 6010, 55)

{

Outlier("General", 0.05);

};

R_Date("GrA-13296", 6050, 50)

{

Outlier("General", 0.05);

};

R_Date("GrM-22776", 6019, 29)

{

Outlier("General", 0.05);

};

R_Date("GrM-26624", 6047, 27)

{

Outlier("General", 0.5);

};

R_Date("GrM-26625", 6080, 27)

{

Outlier("General", 0.5);

};

Date("Occupation in absolute time, de Bruin 2")

{

color="blue";

};

Interval("De Bruin 2, Interval");

Sum("De Bruin 2 sum")

{

};

Span("De Bruin 2 span");

};

Boundary("End de Bruin 2");

Boundary("hiatus");

Boundary("Start de Bruin 3");

Phase("de Bruin 3")

{

R_Date("GRA-64342", 5380, 40)

{

Outlier("General", 0.05);

};

R_Date("GrA-13272", 5900, 50)

{

Outlier("General", 0.05);

};

R_Date("GrM-22775", 5514, 29)

{

Outlier("General", 0.05);

};

R_Date("GrM-22781", 5569, 29)

{

Outlier("General", 0.05);

};

R_Date("GrM-23702", 5500, 45)

{

Outlier("General", 0.05);

};

R_Date("GrA-10950", 5430, 60)

{

Outlier("General", 0.05);

};

R_Date("GrM-23703", 6345, 28)

{

Outlier("General", 0.05);

};

R_Date("GrM-25038", 5541, 24)

{

Outlier("General", 0.05);

};

R_Date("GRA-62951", 5610, 40)

{

Outlier("General", 0.05);

};

Date("Occupation in absolute time, Bruin 3")

{

color="blue";

};

Interval("De Bruin 3, Interval");

Sum("De Bruin 3 sum")

{

};

Span("De Bruin 3 span");

};

Boundary("de Bruin 3");

};

};

S.3 Code for Main model 2 (reference to Fig 8)

Plot()

{

Outlier_Model("General",T(5),U(0,4),"t");

Outlier_Model("IA",Prior("Charcoal_Plus"),U(0,3),"t");

Sequence()

{

Boundary("Start Polderweg 0");

Phase("Polderweg 0")

{

R_Date("GrM-25035", 6492, 29)

{

Outlier("General", 0.05);

};

R_Date("GrM-22769", 6473, 29)

{

Outlier("General", 0.05);

};

Date("Occupation in absolute time, Polderweg 0")

{

color="green";

};

Interval("Polderweg 0, Interval");

};

Boundary("Transition Polderweg 0 to 1");

Phase("Polderweg 1")

{

R_Date("GrA-9798", 6320, 50)

{

Outlier("General",0.05);

};

R_Date("GrA-9803", 6380, 50)

{

Outlier("General",0.05);

};

R_Date("GrA-9799", 6540, 50)

{

Outlier("General",0.05);

};

R_Date("GrA-9797", 6480, 50)

{

Outlier("IA", 1);

};

R_Date("GrA-23896", 6390, 100)

{

Outlier("IA", 1);

};

R_Date("GrM-22774", 6420, 29)

{

Outlier("General", 0.05);

};

R_Date("GrM-22770", 6483, 22)

{

Outlier("General", 0.05);

};

R_Date("GrM-22772", 6298, 29)

{

Outlier("General", 0.05);

};

R_Date("GrM-25033", 6502, 30)

{

Outlier("General", 0.05);

};

R_Date("GrM-23704", 6550, 30)

{

Outlier("General", 0.05);

};

R_Date("GrM-23514", 5383, 26)

{

Outlier("General", 0.05);

};

R_Date("UtC-3075", 6450, 90)

{

Outlier("IA", 1);

};

Date("Occupation in absolute time, Polderweg 1")

{

color="green";

};

Interval("Polderweg 1, Interval");

};

Boundary("End Polderweg 1");

Boundary("Start Polderweg 1/2");

Phase("Polderweg 1/2")

{

R_Date("GrA-9802", 6050, 50)

{

Outlier("General",0.05);

};

R_Date("GrM-26626", 6167, 29)

{

Outlier("General", 0.05);

};

R_Date("GrM-25037", 6223, 29)

{

Outlier("General", 0.05);

};

R_Date("GrM-25036", 6017, 29)

{

Outlier("General", 0.05);

};

Date("Occupation in absolute time, Polderweg 1/2")

{

color="green";

};

Interval("Polderweg 1/2, Interval");

};

Boundary("End Polderweg 1/2");

Boundary("Start Polderweg 2");

Phase("Polderweg 2")

{

R_Date("GrA-9800", 5780, 50)

{

Outlier("General",0.05);

};

R_Date("GrM-23741", 5889, 27)

{

Outlier("General", 0.05);

};

R_Date("GrM-25044", 5905, 29)

{

Outlier("General", 0.05);

};

R_Date("GrM-25045", 5841, 29)

{

Outlier("General", 0.05);

};

Date("Occupation in absolute time, Polderweg 2")

{

color="green";

};

Interval("Polderweg 2, Interval");

};

Boundary("End Polderweg 2");

};

Sequence()

{

Boundary("Start de Bruin 1");

Phase("de Bruin 1")

{

R_Date("GrA-13275", 6420, 50)

{

Outlier("General", 0.05);

};

R_Date("GrM-22778", 6427, 53)

{

Outlier("General", 0.05);

};

R_Date("GrM-22851", 6319, 29)

{

Outlier("General", 0.05);

};

R_Date("GrN-24395", 6310, 90)

{

Outlier("IA", 1);

};

R_Date("GrA-13277", 6100, 50)

{

Outlier("IA", 1);

};

R_Date("GrA-13274", 6130, 50)

{

Outlier("IA", 1);

};

R_Date("GrN-24396", 6310, 60)

{

Outlier("IA", 1);

};

Date("Occupation in absolute time, de Bruin 1")

{

color="blue";

};

Interval("De Bruin 1, Interval");

};

Boundary("End de Bruin 1");

Boundary("Start de Bruin 2");

Phase("de Bruin 2")

{

R_Date("GrM-25043", 6213, 26)

{

Outlier("General", 0.05);

};

R_Date("GrA-14865", 6120, 50)

{

Outlier("General", 0.05);

};

R_Date("GrA-12304", 6170, 50)

{

Outlier("General", 0.05);

};

R_Date("GrM-25040", 6138, 26)

{

Outlier("General", 0.05);

};

R_Date("GrA-15034", 6010, 55)

{

Outlier("General", 0.05);

};

R_Date("GrA-13296", 6050, 50)

{

Outlier("General", 0.05);

};

R_Date("GrM-22776", 6019, 29)

{

Outlier("General", 0.05);

};

R_Date("GrM-26624", 6047, 27)

{

Outlier("General", 0.5);

};

R_Date("GrM-26625", 6080, 27)

{

Outlier("General", 0.5);

};

Date("Occupation in absolute time, de Bruin 2")

{

color="blue";

};

Interval("De Bruin 2, Interval");

};

Boundary("End de Bruin 2");

Boundary("hiatus");

Boundary("Start de Bruin 3");

Phase("de Bruin 3")

{

R_Date("GRA-64342", 5380, 40)

{

Outlier("General", 0.05);

};

R_Date("GrA-13272", 5900, 50)

{

Outlier("General", 0.05);

};

R_Date("GrM-22775", 5514, 29)

{

Outlier("General", 0.05);

};

R_Date("GrM-22781", 5569, 29)

{

Outlier("General", 0.05);

};

R_Date("GrM-23702", 5500, 45)

{

Outlier("General", 0.05);

};

R_Date("GrA-10950", 5430, 60)

{

Outlier("General", 0.05);

};

R_Date("GrM-25038", 5541, 24)

{

Outlier("General", 0.05);

};

R_Date("GrM-23703", 6345, 28)

{

Outlier("General", 0.05);

};

R_Date("GRA-62951", 5610, 40)

{

Outlier("General", 0.05);

};

Date("Occupation in absolute time, Bruin 3")

{

color="blue";

};

Interval("De Bruin 3, Interval");

};

Boundary("de Bruin 3");

};

};

S4. Sensitivity test models

S.4.1. Main model 1, no Outlier model

Options()

{

Resolution=1;

};

Plot()

{

Sequence()

{

Boundary("Start Polderweg 0");

Phase("Polderweg 0")

{

R_Date("GrM-25035", 6492, 29)

{

};

R_Date("GrM-22769", 6473, 29)

{

};

Date("Occupation in absolute time, Polderweg 0")

{

color="green";

};

Interval("Polderweg 0, Interval");

Span("Polderweg 0");

Sum("Polderweg 0 sum")

{

};

};

Boundary("Transition Polderweg 0 to 1");

Phase("Polderweg 1")

{

R_Date("GrA-9798", 6320, 50)

{

};

R_Date("GrA-9803", 6380, 50)

{

};

R_Date("GrA-9799", 6540, 50)

{

};

R_Date("GrA-9797", 6480, 50)

{

};

R_Date("GrA-23896", 6390, 100)

{

};

R_Date("GrM-22774", 6420, 29)

{

};

R_Date("GrM-22770", 6483, 22)

{

};

R_Date("GrM-23704", 6550, 30)

{

};

R_Date("GrM-22772", 6298, 29)

{

};

R_Date("GrM-25033", 6502, 30)

{

};

R_Date("UtC-3075", 6450, 90)

{

};

Date("Occupation in absolute time, Polderweg 1")

{

color="green";

};

Interval("Polderweg 1, Interval");

Sum("Polderweg 1 sum")

{

};

Span("Polderweg 1 span");

};

Boundary("End Polderweg 1");

Boundary("Start Polderweg 1/2");

Phase("Polderweg 1/2")

{

R_Date("GrA-9802", 6050, 50)

{

};

R_Date("GrM-26626", 6167, 29)

{

};

R_Date("GrM-25037", 6223, 29)

{

};

R_Date("GrM-25036", 6017, 29)

{

};

Date("Occupation in absolute time, Polderweg 1/2")

{

color="green";

};

Interval("Polderweg 1/2, Interval");

Sum("Polderweg 1/2 sum")

{

};

Span("Polderweg 1/2 span");

};

Boundary("End Polderweg 1/2");

Boundary("Start Polderweg 2");

Phase("Polderweg 2")

{

R_Date("GrA-9800", 5780, 50)

{

};

R_Date("GrM-23741", 5889, 27)

{

};

R_Date("GrM-25044", 5905, 29)

{

};

R_Date("GrM-25045", 5841, 29)

{

};

Date("Occupation in absolute time, Polderweg 2")

{

color="green";

};

Interval("Polderweg 2, Interval");

Sum("Polderweg 2 sum")

{

};

Span("Polderweg 2 span");

};

Boundary("End Polderweg 2");

};

Sequence()

{

Boundary("Start de Bruin 1");

Phase("de Bruin 1")

{

R_Date("GrA-13275", 6420, 50)

{

};

R_Date("GrM-22778", 6427, 53)

{

};

R_Date("GrM-22851", 6319, 29)

{

};

R_Date("GrN-24395", 6310, 90)

{

};

R_Date("GrA-13277", 6100, 50)

{

};

R_Date("GrA-13274", 6130, 50)

{

};

R_Date("GrN-24396", 6310, 60)

{

};

Date("Occupation in absolute time, de Bruin 1")

{

color="blue";

};

Interval("De Bruin 1, Interval");

Sum("De Bruin 1 sum")

{

};

Span("De Bruin 1 span");

};

Boundary("End de Bruin 1");

Boundary("Start de Bruin 2");

Phase("de Bruin 2")

{

R_Date("GrM-25043", 6213, 26)

{

};

R_Date("GrA-14865", 6120, 50)

{

};

R_Date("GrA-12304", 6170, 50)

{

};

R_Date("GrM-25040", 6138, 26)

{

};

R_Date("GrA-15034", 6010, 55)

{

};

R_Date("GrA-13296", 6050, 50)

{

};

R_Date("GrM-22776", 6019, 29)

{

};

R_Date("GrM-26624", 6047, 27)

{

};

R_Date("GrM-26625", 6080, 27)

{

};

R_Date("GrA-14864", 5685, 50);

R_Date("GrA-13278", 5730, 50);

Date("Occupation in absolute time, de Bruin 2")

{

color="blue";

};

Interval("De Bruin 2, Interval");

Sum("De Bruin 2 sum")

{

};

Span("De Bruin 2 span");

};

Boundary("End de Bruin 2");

Boundary("hiatus");

Boundary("Start de Bruin 3");

Phase("de Bruin 3")

{

R_Date("GRA-64342", 5380, 40)

{

};

R_Date("GrM-22775", 5514, 29)

{

};

R_Date("GrM-22781", 5569, 29)

{

};

R_Date("GrA-13272", 5900, 50)

{

};

R_Date("GrM-23702", 5500, 45)

{

};

R_Date("GrA-10950", 5430, 60)

{

};

R_Date("GrM-25038", 5541, 24)

{

};

R_Date("GRA-62951", 5610, 40)

{

};

Date("Occupation in absolute time, Bruin 3")

{

color="blue";

};

Interval("De Bruin 3, Interval");

Sum("De Bruin 3 sum")

{

};

Span("De Bruin 3 span");

};

Boundary("de Bruin 3");

};

};

S.4.2. Main model 1, no Outlier model, excluded outliers (> 60% Overall Agreement)

Options()

{

Resolution=1;

};

Plot()

{

Sequence()

{

Boundary("Start Polderweg 0");

Phase("Polderweg 0")

{

R_Date("GrM-25035", 6492, 29)

{

};

R_Date("GrM-22769", 6473, 29)

{

};

Date("Occupation in absolute time, Polderweg 0")

{

color="green";

};

Interval("Polderweg 0, Interval");

Span("Polderweg 0");

Sum("Polderweg 0 sum")

{

};

};

Boundary("Transition Polderweg 0 to 1");

Phase("Polderweg 1")

{

R_Date("GrA-9798", 6320, 50)

{

};

R_Date("GrA-9803", 6380, 50)

{

};

R_Date("GrA-9799", 6540, 50)

{

};

R_Date("GrA-9797", 6480, 50)

{

};

R_Date("GrA-23896", 6390, 100)

{

};

R_Date("GrM-22774", 6420, 29)

{

};

R_Date("GrM-22770", 6483, 22)

{

};

R_Date("GrM-22772", 6298, 29)

{

};

R_Date("GrM-25033", 6502, 30)

{

};

R_Date("UtC-3075", 6450, 90)

{

};

Date("Occupation in absolute time, Polderweg 1")

{

color="green";

};

Interval("Polderweg 1, Interval");

Sum("Polderweg 1 sum")

{

};

Span("Polderweg 1 span");

};

Boundary("End Polderweg 1");

Boundary("Start Polderweg 1/2");

Phase("Polderweg 1/2")

{

R_Date("GrA-9802", 6050, 50)

{

};

R_Date("GrM-26626", 6167, 29)

{

};

R_Date("GrM-25037", 6223, 29)

{

};

R_Date("GrM-25036", 6017, 29)

{

};

Date("Occupation in absolute time, Polderweg 1/2")

{

color="green";

};

Interval("Polderweg 1/2, Interval");

Sum("Polderweg 1/2 sum")

{

};

Span("Polderweg 1/2 span");

};

Boundary("End Polderweg 1/2");

Boundary("Start Polderweg 2");

Phase("Polderweg 2")

{

R_Date("GrA-9800", 5780, 50)

{

};

R_Date("GrM-23741", 5889, 27)

{

};

R_Date("GrM-25044", 5905, 29)

{

};

R_Date("GrM-25045", 5841, 29)

{

};

Date("Occupation in absolute time, Polderweg 2")

{

color="green";

};

Interval("Polderweg 2, Interval");

Sum("Polderweg 2 sum")

{

};

Span("Polderweg 2 span");

};

Boundary("End Polderweg 2");

};

Sequence()

{

Boundary("Start de Bruin 1");

Phase("de Bruin 1")

{

R_Date("GrA-13275", 6420, 50)

{

};

R_Date("GrM-22778", 6427, 53)

{

};

R_Date("GrM-22851", 6319, 29)

{

};

R_Date("GrN-24395", 6310, 90)

{

};

R_Date("GrA-13277", 6100, 50)

{

};

R_Date("GrA-13274", 6130, 50)

{

};

R_Date("GrN-24396", 6310, 60)

{

};

Date("Occupation in absolute time, de Bruin 1")

{

color="blue";

};

Interval("De Bruin 1, Interval");

Sum("De Bruin 1 sum")

{

};

Span("De Bruin 1 span");

};

Boundary("End de Bruin 1");

Boundary("Start de Bruin 2");

Phase("de Bruin 2")

{

R_Date("GrM-25043", 6213, 26)

{

};

R_Date("GrA-14865", 6120, 50)

{

};

R_Date("GrA-12304", 6170, 50)

{

};

R_Date("GrM-25040", 6138, 26)

{

};

R_Date("GrA-15034", 6010, 55)

{

};

R_Date("GrA-13296", 6050, 50)

{

};

R_Date("GrM-22776", 6019, 29)

{

};

R_Date("GrM-26624", 6047, 27)

{

};

R_Date("GrM-26625", 6080, 27)

{

};

R_Date("GrA-14864", 5685, 50);

R_Date("GrA-13278", 5730, 50);

Date("Occupation in absolute time, de Bruin 2")

{

color="blue";

};

Interval("De Bruin 2, Interval");

Sum("De Bruin 2 sum")

{

};

Span("De Bruin 2 span");

};

Boundary("End de Bruin 2");

Boundary("hiatus");

Boundary("Start de Bruin 3");

Phase("de Bruin 3")

{

R_Date("GRA-64342", 5380, 40)

{

};

R_Date("GrM-22775", 5514, 29)

{

};

R_Date("GrM-22781", 5569, 29)

{

};

R_Date("GrM-23702", 5500, 45)

{

};

R_Date("GrA-10950", 5430, 60)

{

};

R_Date("GrM-25038", 5541, 24)

{

};

R_Date("GRA-62951", 5610, 40)

{

};

Date("Occupation in absolute time, Bruin 3")

{

color="blue";

};

Interval("De Bruin 3, Interval");

Sum("De Bruin 3 sum")

{

};

Span("De Bruin 3 span");

};

Boundary("de Bruin 3");

};

};

**S.4.3. Main model 1 with third-grade legacy dates (command After)**

Options()

{

Resolution=1;

};

Plot()

{

Outlier_Model("General",T(5),U(0,4),"t");

Outlier_Model("IA",Prior("Charcoal_Plus"),U(0,3),"t");

Sequence()

{

Boundary("Start Polderweg 0");

Phase("Polderweg 0")

{

R_Date("GrM-25035", 6492, 29)

{

Outlier("General", 0.05);

};

R_Date("GrM-22769", 6473, 29)

{

Outlier("General", 0.05);

};

After("Reservoir effect")

{

R_Date("GrA-9804", 6820 , 50)

{

Outlier("General", 0.05);

};

};

Interval("Polderweg 0, Interval");

Span("Polderweg_0");

Sum("Polderweg_0 sum")

{

};

Date("Occupation in absolute time, Polderweg 0")

{

color="green";

};

};

Boundary("Transition Polderweg 0 to 1");

Phase("Polderweg 1")

{

R_Date("GrA-9798", 6320, 50)

{

Outlier("General",0.05);

};

R_Date("GrA-9803", 6380, 50)

{

Outlier("General",0.05);

};

R_Date("GrA-9799", 6540, 50)

{

Outlier("General",0.05);

};

R_Date("GrA-9797", 6480, 50)

{

Outlier("IA", 1);

};

R_Date("GrA-23896", 6390, 100)

{

Outlier("IA", 1);

};

R_Date("GrM-22774", 6420, 29)

{

Outlier("General", 0.05);

};

R_Date("GrM-22770", 6483, 22)

{

Outlier("General", 0.05);

};

R_Date("GrM-22772", 6298, 29)

{

Outlier("General", 0.05);

};

R_Date("GrM-25033", 6502, 30)

{

Outlier("General", 0.05);

};

R_Date("GrM-23704", 6550, 30)

{

Outlier("General", 0.05);

};

R_Date("UtC-3075", 6450, 90)

{

Outlier("IA", 1);

};

After("Reservoir effect")

{

R_Date("GrA-10902", 5880 , 60)

{

Outlier("General", 0.05);

};

};

After("Reservoir effect")

{

R_Date("GrA-9807", 6650 , 50)

{

Outlier("General", 0.05);

};

};

Interval("Polderweg 1, Interval");

Span("Polderweg_1");

Sum("Polderweg_1 sum")

{

};

Date("Occupation in absolute time, Polderweg 1")

{

color="green";

};

};

Boundary("End Polderweg 1");

Boundary("Start Polderweg 1/2");

Phase("Polderweg 1/2")

{

R_Date("GrA-9802", 6050, 50)

{

Outlier("General",0.05);

};

R_Date("GrM-26626", 6167, 29)

{

Outlier("General", 0.05);

};

R_Date("GrM-25037", 6223, 29)

{

Outlier("General", 0.05);

};

R_Date("GrM-25036", 6017, 29)

{

Outlier("General", 0.05);

};

Date("Occupation in absolute time, Polderweg 1/2")

{

color="green";

};

Interval("Polderweg 1/2, Interval");

Span("Polderweg_1-2");

Sum("Polderweg_1/2 sum")

{

};

};

Boundary("End Polderweg 1/2");

Boundary("Start Polderweg 2");

Phase("Polderweg 2")

{

R_Date("GrA-9800", 5780, 50)

{

Outlier("General",0.05);

};

R_Date("GrM-23741", 5889, 27)

{

Outlier("General", 0.05);

};

R_Date("GrM-25044", 5905, 29)

{

Outlier("General", 0.05);

};

R_Date("GrM-25045", 5841, 29)

{

Outlier("General", 0.05);

};

After("Reservoir effect")

{

R_Date("GrA-11829", 6130 , 50)

{

Outlier("General", 0.05);

};

};

After("Reservoir effect")

{

R_Date("GrA-11841", 6140 , 50)

{

Outlier("General", 0.05);

};

};

After("Reservoir effect")

{

R_Date("GrA-11830", 6170 , 50)

{

Outlier("General", 0.05);

};

};

Date("Occupation in absolute time, Polderweg 2")

{

color="green";

};

Sum("Polderweg_2 sum")

{

};

Span("Polderweg_2");

Interval("Polderweg 2, Interval");

};

Boundary("End Polderweg 2");

};

Sequence()

{

Boundary("Start de Bruin 1");

Phase("de Bruin 1")

{

R_Date("GrA-13275", 6420, 50)

{

Outlier("General", 0.05);

};

R_Date("GrM-22778", 6427, 53)

{

Outlier("General", 0.05);

};

R_Date("GrM-22851", 6319, 29)

{

Outlier("General", 0.05);

};

R_Date("GrN-24395", 6310, 90)

{

Outlier("IA", 1);

};

R_Date("GrA-13277", 6100, 50)

{

Outlier("IA", 1);

};

R_Date("GrA-13274", 6130, 50)

{

Outlier("IA", 1);

};

R_Date("GrN-24396", 6310, 60)

{

Outlier("IA", 1);

};

Date("Occupation in absolute time, de Bruin 1")

{

color="blue";

};

Interval("De Bruin 1, Interval");

Span("De Bruin_1");

Sum("De Bruin_1 sum")

{

};

};

Boundary("End de Bruin 1");

Boundary("Start de Bruin 2");

Phase("de Bruin 2")

{

R_Date("GrM-25043", 6213, 26)

{

Outlier("General", 0.05);

};

R_Date("GrA-14865", 6120, 50)

{

Outlier("General", 0.05);

};

R_Date("GrA-12304", 6170, 50)

{

Outlier("General", 0.05);

};

R_Date("GrM-25040", 6138, 26)

{

Outlier("General", 0.05);

};

R_Date("GrA-15034", 6010, 55)

{

Outlier("General", 0.05);

};

R_Date("GrA-13296", 6050, 50)

{

Outlier("General", 0.05);

};

R_Date("GRA-62951", 5610, 40)

{

Outlier("General", 0.05);

};

R_Date("GrA-13278", 5730, 50)

{

Outlier("General", 0.05);

};

R_Date("GrA-14864", 5685, 50)

{

Outlier("General", 0.05);

};

R_Date("GrM-22776", 6019, 29)

{

Outlier("General", 0.05);

};

R_Date("GrM-26624", 6047, 27)

{

Outlier("General", 0.05);

};

R_Date("GrM-26625", 6080, 27)

{

Outlier("General", 0.05);

};

After("Reservoir effect")

{

R_Date("GrA-13315", 6070, 50)

{

Outlier("General", 0.05);

};

};

After("Reservoir effect")

{

R_Date("GrA-13313", 6090, 50)

{

Outlier("General", 0.05);

};

};

After("Reservoir effect")

{

R_Date("GrA-13318", 6100, 50)

{

Outlier("General", 0.05);

};

};

Date("Occupation in absolute time, de Bruin 2")

{

color="blue";

};

Span("De Bruin_2");

Sum("De Bruin_2 sum")

{

};

Interval("De Bruin 2, Interval");

};

Boundary("End de Bruin 2");

Boundary("hiatus");

Boundary("Start de Bruin 3");

Phase("de Bruin 3")

{

R_Date("GRA-64342", 5380, 40)

{

Outlier("General", 0.05);

};

R_Date("GrA-13272", 5900, 50)

{

Outlier("General", 0.05);

};

R_Date("GrM-22775", 5514, 29)

{

Outlier("General", 0.05);

};

R_Date("GrM-22781", 5569, 29)

{

Outlier("General", 0.05);

};

R_Date("GrM-23702", 5500, 45)

{

Outlier("General", 0.05);

};

R_Date("GrA-10950", 5430, 60)

{

Outlier("General", 0.05);

};

R_Date("GrM-25038", 5541, 24)

{

Outlier("General", 0.05);

};

R_Date("GrM-23703", 6345, 28)

{

Outlier("General", 0.05);

};

After("Reservoir effect")

{

R_Date("GrA-13320", 5730, 50)

{

Outlier("General", 0.05);

};

};

After("Reservoir effect")

{

R_Date("GrA-GrA-13317", 5880, 50)

{

Outlier("General", 0.05);

};

};

Sum("De Bruin_3 sum")

{

};

Date("Occupation in absolute time, Bruin 3")

{

color="blue";

};

Interval("De Bruin 3, Interval");

Span("De Bruin_3");

};

Boundary("de Bruin 3");

};

};

**S.4.4. Model De Bruin with simulations (with Outlier model)**

Plot()

{

Outlier_Model("General",T(5),U(0,4),"t");

Outlier_Model("IA",Prior("Charcoal_Plus"),U(0,3),"t");

Sequence()

{

Boundary("Start De Bruin 1");

Phase("De Bruin 1")

{

R_Date("GrA-13275", 6420, 50)

{

Outlier("General", 0.05);

};

R_Date("GrM-22778", 6427, 53)

{

Outlier("General", 0.05);

};

R_Date("GrM-22851", 6319, 29)

{

Outlier("General", 0.05);

};

R_Date("GrN-24395", 6310, 90)

{

Outlier("IA", 1);

};

R_Date("GrA-13277", 6100, 50)

{

Outlier("IA", 1);

};

R_Date("GrA-13274", 6130, 50)

{

Outlier("IA", 1);

};

R_Date("GrN-24396", 6310, 60)

{

Outlier("IA", 1);

};

Date("Duration De Bruin 1")

{

color="blue";

};

};

Boundary("End De Bruin 1");

Boundary("Start De Bruin 2");

Phase("De Bruin 2")

{

R_Date("GrA-14865", 6120, 50)

{

Outlier("General", 0.05);

};

R_Date("GrA-12304", 6170, 50)

{

Outlier("General", 0.05);

};

R_Date("GrM-25040", 6138, 26)

{

Outlier("General", 0.05);

};

R_Date("GrM-25043", 6213, 26)

{

Outlier("General", 0.05);

};

R_Date("GrA-15034", 6010, 55)

{

Outlier("General", 0.05);

};

R_Date("GrA-13296", 6050, 50)

{

Outlier("General", 0.05);

};

R_Date("GRA-62951", 5610, 40)

{

Outlier("General", 0.05);

};

R_Date("GrA-13278", 5730, 50)

{

Outlier("General", 0.05);

};

R_Date("GrA-14864", 5685, 50)

{

Outlier("General", 0.05);

};

R_Date("GrM-22776", 6019, 29)

{

Outlier("General", 0.05);

};

R_Date("GrM-26624", 6047, 27)

{

Outlier("General", 0.5);

};

R_Date("GrM-26625", 6080, 27)

{

Outlier("General", 0.5);

};

R_Simulate("Sim1", -4850, 27)

{

Outlier("General", 0.05);

};

R_Simulate("Sim_2", -4850, 27)

{

Outlier("General", 0.05);

};

R_Simulate("Sim 3", -4865, 27)

{

Outlier("General", 0.05);

};

R_Simulate("Sim 4", -4855, 27)

{

Outlier("General", 0.05);

};

R_Simulate("Sim 5", -4800, 27)

{

Outlier("General", 0.05);

};

R_Simulate("Sim 6", -4805, 27)

{

Outlier("General", 0.05);

};

R_Simulate("Sim 7", -4825, 27)

{

Outlier("General", 0.05);

};

R_Simulate("Sim 8", -4805, 27)

{

Outlier("General", 0.05);

};

Date("Duration De Bruin 2")

{

color="blue";

};

R_Simulate("Sim 9", -4800, 27)

{

Outlier("General", 0.05);

};

R_Simulate("Sim 10", -4795, 27)

{

Outlier("General", 0.05);

};

R_Simulate("Sim 11", -4825, 27)

{

Outlier("General", 0.05);

};

R_Simulate("Sim 12", -4800, 27)

{

Outlier("General", 0.05);

};

R_Simulate("Sim 13", -4800, 27)

{

Outlier("General", 0.05);

};

R_Simulate("Sim 14", -4800, 27)

{

Outlier("General", 0.05);

};

R_Simulate("Sim 15", -4800, 27)

{

Outlier("General", 0.05);

};

R_Simulate("Sim 16", -4850, 27)

{

Outlier("General", 0.05);

};

R_Simulate("Sim 17", -4800, 27)

{

Outlier("General", 0.05);

};

};

Boundary("End De Bruin 2");

Boundary("Start De Bruin 3");

Phase("De Bruin 3")

{

R_Date("GRA-64342", 5380, 40)

{

Outlier("General", 0.05);

};

R_Date("GrA-13272", 5900, 50)

{

Outlier("General", 0.05);

};

R_Date("GrM-22775", 5514, 29)

{

Outlier("General", 0.05);

};

R_Date("GrM-22781", 5569, 29)

{

Outlier("General", 0.05);

};

R_Date("GrM-23702", 5500, 45)

{

Outlier("General", 0.05);

};

R_Date("GrA-10950", 5430, 60)

{

Outlier("General", 0.05);

};

R_Date("GrM-25038", 5541, 24)

{

Outlier("General", 0.05);

};

Date("Duration De Bruin 3")

{

color="blue";

};

};

Boundary("End De Bruin 3");

R_Date("GrM-23703", 6345, 28)

{

Outlier("General", 0.05);

};

};

};

**S.4.5.** **Model with solely new (EDAN) dates, no Outlier Analysis**

Options()

{

Resolution=1;

};

Plot()

{

Sequence()

{

Boundary("Start Polderweg 0");

Phase("Polderweg 0")

{

R_Date("GrM-25035", 6492, 29)

{

};

R_Date("GrM-22769", 6473, 29)

{

};

Date("Occupation in absolute time, Polderweg 0")

{

color="green";

};

Interval("Polderweg 0, Interval");

Span("Polderweg 0");

Sum("Polderweg 0 sum")

{

};

};

Boundary("Transition Polderweg 0 to 1");

Phase("Polderweg 1")

{

R_Date("GrM-22774", 6420, 29)

{

};

R_Date("GrM-22770", 6483, 22)

{

};

R_Date("GrM-22772", 6298, 29)

{

};

R_Date("GrM-25033", 6502, 30)

{

};

R_Date("GrM-23704", 6550, 30)

{

};

Date("Occupation in absolute time, Polderweg 1")

{

color="green";

};

Interval("Polderweg 1, Interval");

Sum("Polderweg 1 sum")

{

};

Span("Polderweg 1 span");

};

Boundary("End Polderweg 1");

Boundary("Start Polderweg 1/2");

Phase("Polderweg 1/2")

{

R_Date("GrM-26626", 6167, 29)

{

};

R_Date("GrM-25037", 6223, 29)

{

};

R_Date("GrM-25036", 6017, 29)

{

};

Date("Occupation in absolute time, Polderweg 1/2")

{

color="green";

};

Interval("Polderweg 1/2, Interval");

Sum("Polderweg 1/2 sum")

{

};

Span("Polderweg 1/2 span");

};

Boundary("End Polderweg 1/2");

Boundary("Start Polderweg 2");

Phase("Polderweg 2")

{

R_Date("GrM-23741", 5889, 27)

{

};

R_Date("GrM-25044", 5905, 29)

{

};

R_Date("GrM-25045", 5841, 29)

{

};

Date("Occupation in absolute time, Polderweg 2")

{

color="green";

};

Interval("Polderweg 2, Interval");

Sum("Polderweg 2 sum")

{

};

Span("Polderweg 2 span");

};

Boundary("End Polderweg 2");

};

Sequence()

{

Boundary("Start de Bruin 1");

Phase("de Bruin 1")

{

R_Date("GrM-22778", 6427, 53)

{

};

R_Date("GrM-22851", 6319, 29)

{

};

Date("Occupation in absolute time, de Bruin 1")

{

color="blue";

};

Interval("De Bruin 1, Interval");

Sum("De Bruin 1 sum")

{

};

Span("De Bruin 1 span");

};

Boundary("End de Bruin 1");

Boundary("Start de Bruin 2");

Phase("de Bruin 2")

{

R_Date("GrM-25043", 6213, 26)

{

};

R_Date("GrM-25040", 6138, 26)

{

};

R_Date("GrM-22776", 6019, 29)

{

};

R_Date("GrM-26624", 6047, 27)

{

};

R_Date("GrM-26625", 6080, 27)

{

};

Date("Occupation in absolute time, de Bruin 2")

{

color="blue";

};

Interval("De Bruin 2, Interval");

Sum("De Bruin 2 sum")

{

};

Span("De Bruin 2 span");

};

Boundary("End de Bruin 2");

Boundary("hiatus");

Boundary("Start de Bruin 3");

Phase("de Bruin 3")

{

R_Date("GrM-22775", 5514, 29)

{

};

R_Date("GrM-22781", 5569, 29)

{

};

R_Date("GrM-23702", 5500, 45)

{

};

R_Date("GrM-25038", 5541, 24)

{

};

Date("Occupation in absolute time, Bruin 3")

{

color="blue";

};

Interval("De Bruin 3, Interval");

Sum("De Bruin 3 sum")

{

};

Span("De Bruin 3 span");

};

Boundary("de Bruin 3");

};

};

**S.4.6. Polderweg, Model without Outlier Analysis, merged Phase 0 and 1**

Options()

{

Resolution=1;

};

Plot()

{

Sequence()

{

Boundary("Start Polderweg 1");

Phase("Polderweg 1")

{

R_Date("GrA-9798", 6320, 50)

{

};

R_Date("GrA-9803", 6380, 50)

{

};

R_Date("GrA-9799", 6540, 50)

{

};

R_Date("GrA-9797", 6480, 50)

{

};

R_Date("GrA-23896", 6390, 100)

{

};

R_Date("GrM-22774", 6420, 29)

{

};

R_Date("GrM-22770", 6483, 22)

{

};

R_Date("GrM-22772", 6298, 29)

{

};

R_Date("GrM-25033", 6502, 30)

{

};

R_Date("UtC-3075", 6450, 90)

{

};

R_Date("GrM-25035", 6492, 29)

{

};

R_Date("GrM-22769", 6473, 29)

{

};

Date("Duration Polderweg 1")

{

color="green";

};

};

Boundary("End Polderweg 1");

Boundary("Start Polderweg 1/2");

Phase("Polderweg 1/2")

{

R_Date("GrA-9802", 6050, 50)

{

};

R_Date("GrM-26626", 6167, 29)

{

};

R_Date("GrM-25037", 6223, 29)

{

};

R_Date("GrM-25036", 6017, 29)

{

};

Date("Duration Polderweg 1/2")

{

color="green";

};

};

Boundary("End Polderweg 1/2");

Boundary("Start Polderweg 2");

Phase("Polderweg 2")

{

R_Date("GrA-9800", 5780, 50)

{

};

R_Date("GrM-23741", 5889, 27)

{

};

R_Date("GrM-25044", 5905, 29)

{

};

R_Date("GrM-25045", 5841, 29)

{

};

Date("Duration Polderweg 2")

{

color="green";

};

};

Boundary("End Polderweg 2");

};

};

S.5. Plot with modelled probability densities of Hardinxveld sites, with Outlier Analysis; included third-grade legacy dates


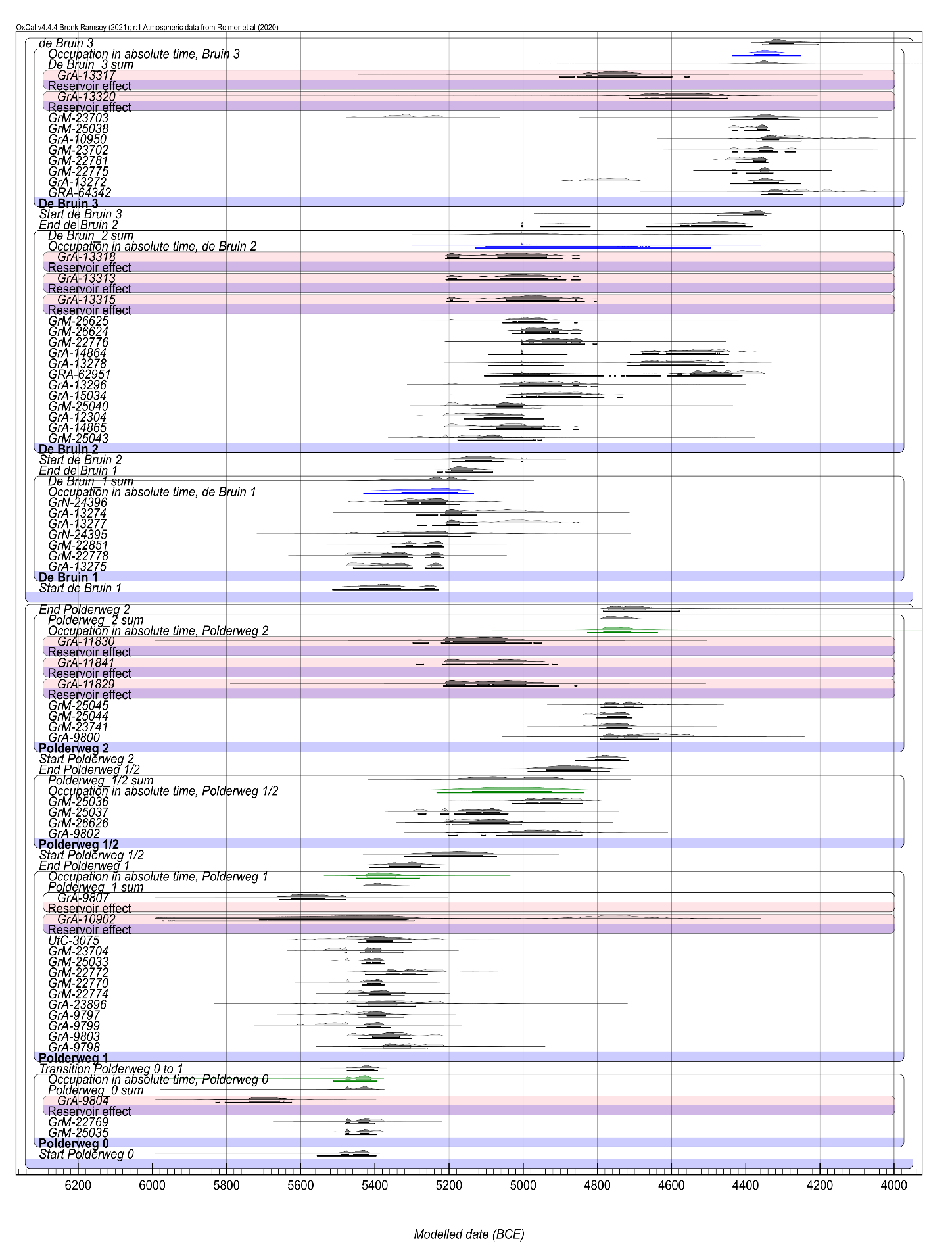


Literature:

Bakels CC, van Beurden LM, Vernimmen TJJ. Archeobotanie. In: Hardinxveld-Giessendam De Bruin, een kampplaats uit het laat-mesolithicum en het begin van de Swifterbantcultuur in de Rijn/Maasdelta, 5500-4450 v Chr 200. Amersfoort: Archol BV; 2001. p. 369–433. Available from: <https://doi.org/10.17026/dans-zqw-7dvd>

Çakırlar C, Breider, Rianne K Francis, Cohen, Kim M. R Daan CM. Dealing with domestic animals in the fith millennium cal BC Dutch wetlands:new insights from old Swifterbant assemblages. In: Farmers at the Frontier: A Pan European Perspective on Neolithisation. Oxbow Books; 2020. p. 263–87.

Kooijmans LP, Mol J. Stratigrafie, chronologie en fasering. In: Hardinxveld-Giessendam Polderweg, Een mesolithisch jachtkamp in het rivierengebied (5500-5000 v Chr ). Amersfoort; 2001. p. 57–73. Available from: https://doi.org/10.17026/dans-zqw-7dvd

Mol J, Kooijmans LP. Stratigrafie, chronologie en fasering. In: Hardinxveld-Giessendam De Bruin, een kampplaats uit het laat-mesolithicum en het begin van de Swifterbantcultuur in de Rijn/Maasdelta, 5500-4450 v Chr. Amersfoort: Archol BV; 2001. p. 57–73.
